# Spatially Resolved Band Gap and Dielectric Function in Two-Dimensional Materials from Electron Energy Loss Spectroscopy

**DOI:** 10.1021/acs.jpca.1c09566

**Published:** 2022-02-15

**Authors:** Abel Brokkelkamp, Jaco ter Hoeve, Isabel Postmes, Sabrya E. van Heijst, Louis Maduro, Albert V. Davydov, Sergiy Krylyuk, Juan Rojo, Sonia Conesa-Boj

**Affiliations:** † Kavli Institute of Nanoscience, Delft University of Technology, 2628CJ Delft, The Netherlands; ‡ Nikhef Theory Group, Science Park 105, 1098 XG Amsterdam, The Netherlands; § Physics and Astronomy, Vrije Universiteit Amsterdam, 1081 HV Amsterdam, The Netherlands; ∥ Materials Science and Engineering Division, National Institute of Standards and Technology, Gaithersburg, Maryland 20899, United States

## Abstract

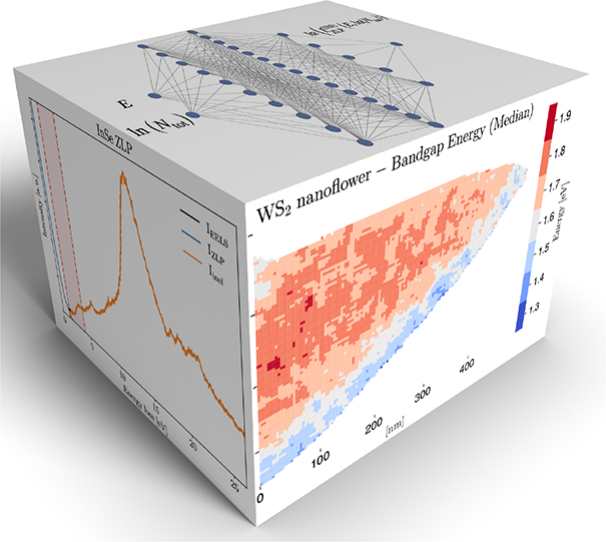

The electronic properties of two-dimensional (2D) materials depend sensitively on the underlying atomic arrangement down to the monolayer level. Here we present a novel strategy for the determination of the band gap and complex dielectric function in 2D materials achieving a spatial resolution down to a few nanometers. This approach is based on machine learning techniques developed in particle physics and makes possible the automated processing and interpretation of spectral images from electron energy loss spectroscopy (EELS). Individual spectra are classified as a function of the thickness with *K*-means clustering, and then used to train a deep-learning model of the zero-loss peak background. As a proof of concept we assess the band gap and dielectric function of InSe flakes and polytypic WS_2_ nanoflowers and correlate these electrical properties with the local thickness. Our flexible approach is generalizable to other nanostructured materials and to higher-dimensional spectroscopies and is made available as a new release of the open-source EELSfitter framework.

## Introduction

Accelerating ongoing investigations of two-dimensional (2D) materials, whose electronic properties depend on the underlying atomic arrangement down to the single-monolayer level, demands novel approaches able to map this sensitive interplay with the highest possible resolution. In this context, electron energy loss spectroscopy (EELS) analyses in scanning transmission electron microscopy (STEM) provide access to a plethora of structural, chemical, and local electronic information,^1−5^ from the thickness and composition to the band gap and complex dielectric function. Crucially, EELS-STEM measurements can be acquired as spectral images (SI), whereby each pixel corresponds to a highly localized region of the specimen. The combination of the excellent spatial and energy resolution provided by state-of-the-art STEM-EELS analyses^6−8^ makes possible deploying EELS-SI as a powerful and versatile tool to realize the spatially resolved simultaneous characterization of structural and electric properties in nanomaterials. Such an approach is complementary to related techniques such as cathodoluminescence in STEM (STEM-CL), which, however, is restricted to radiative processes, while STEM-EELS probes both radiative and nonradiative processes.^9−11^

Fully exploiting this potential requires tackling two main challenges. First, each SI is composed by up to tens of thousands of individual spectra, which need to be jointly processed in a coherent manner. Second, each spectrum is affected by a different zero-loss peak (ZLP) background,^12^ which depends in particular with the local thickness.^5,13^ A robust subtraction of this ZLP is instrumental to interpret the low-loss region (energy loss less than or close to a few electronvolts) in terms of phenomena^11^ such as phonons, excitons, intra- and interband transitions, and to determine the local band gap. Furthermore, one should avoid the pitfalls of traditional ZLP subtraction methods^14−22^ such as the need to specify an *ad hoc* parametric functional dependence.

In this work we bypass these challenges by presenting a novel strategy for the spatially resolved determination of the band gap and complex dielectric function in nanostructured materials from EELS-SI. Our approach is based on machine learning (ML) techniques originally developed in particle physics^23−25^ and achieves a spatial resolution down to a few nanometers. Individual EEL spectra are first classified as a function of the thickness with *K*-means clustering and subsequently used to train a deep-learning model of the dominant ZLP background.^26^ The resultant ZLP-subtracted SI are amenable to theoretical processing, in particular in terms of Fourier transform deconvolution and Kramers–Kronig analyses, leading to a precise determination of relevant structural and electronic properties at the nanoscale.

As a proof of concept we apply our strategy to the determination of the band gap and the complex dielectric function in two representative van der Waals materials, InSe flakes and polytypic WS_2_ nanoflowers.^27^ Both electronic properties are evaluated across the whole specimen and can be correlated among them, e.g., to assess the interplay between the band gap energy or the location of plasmonic resonances with the local thickness. Our approach is amenable to generalization to other families of nanostructured materials, is suitable for application to higher-dimensional data sets such as momentum-resolved EELS, and is made available as a new release of the EELSfitter open-source framework.^26^

## Computational Details

**Figure 1:**
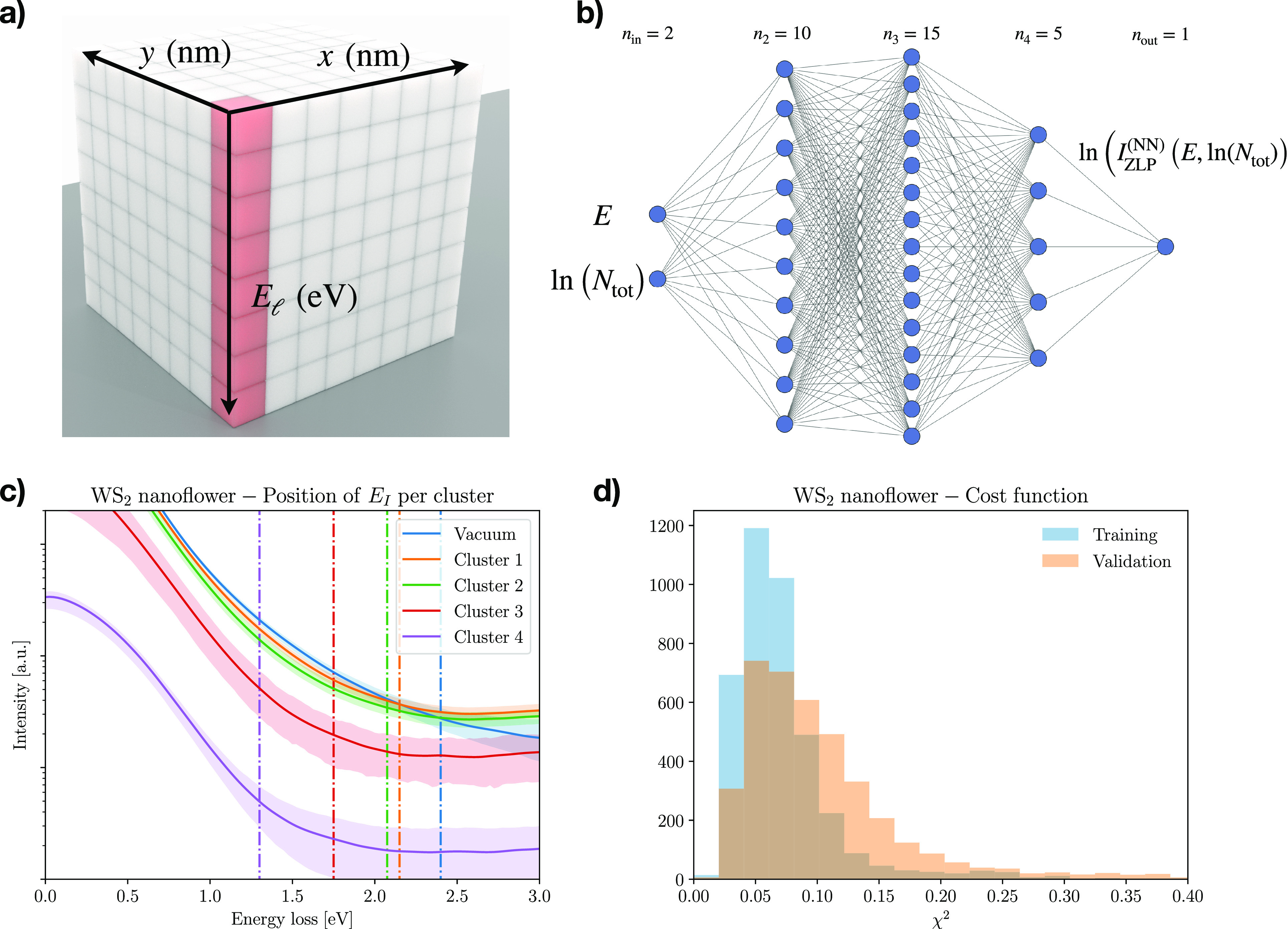
(a) Schematic data cube representing EELS-SI measurements, with two directions labeling the location across the specimen and the third one the energy loss and whose entries are the total intensity  in eq 1. (b) The network architecture parametrizing the ZLP. The input neurons are the energy loss *E* and the integrated intensity *N*_tot_, while the output neuron is the model prediction for the ZLP intensity. (c) The *E*_I_ hyperparameter defines the model training region and is determined from the first derivative d*I*_EELS_/d*E* in each thickness cluster. (d) The training and validation cost function *C*_ZLP_, eq 3, evaluated over 5000 models. Both panels b and c correspond to the WS_2_ nanoflower specimen.

Spectral images in EELS-STEM are constituted by a large number, up to , of individual spectra acquired across the analyzed specimen. They combine the excellent spatial resolution, , achievable with STEM with the competitive energy resolution, , offered by monochromated EELS. From these EELS-SI it is possible to evaluate key quantities such as the local thickness, the band gap energy and type, and the complex dielectric function, provided one first subtracts the ZLP background which dominates the low-loss region of the EEL spectra. The information provided by an EELS-SI can hence be represented by a three-dimensional data cube (Figure 1a)
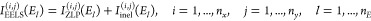
1where *I*_EELS_^(*i*,*j*)^ indicates the total recorded intensity for an electron energy loss  corresponding to the position (*i*, *j*) in the specimen. This intensity receives contributions from the inelastic scatterings off the electrons in the specimen, *I*_inel_, and from the ZLP arising from elastic scatterings and instrumental broadening, *I*_ZLP_. In order to reduce statistical fluctuations, it is convenient to combine the information from neighboring spectra using the pooling procedure described in the section S1 in the Supporting Information.

Since the ZLP intensity depends strongly on the local thickness of the specimen, first of all we group individual spectra as a function of their thickness by means of unsupervised machine learning, specifically by means of the *K*-means clustering algorithm presented in section S1. The cluster assignments are determined from the minimization of a cost function, *C*_Kmeans_, defined in thickness space
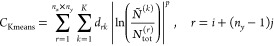
2with *d*_*rk*_ being a binary assignment variable, equal to 1 if *r* belongs to cluster *k* (*d*_*rk*_ = 1 for *r* ∈ *T*_*k*_) and zero otherwise, and with the exponent satisfying *p* > 0. Here *N*_tot_^(*r*)^ represents the integral of *I*_EELS_^(*i*,*j*)^ over the measured range of energy losses, which provides a suitable proxy for the local thickness, and *Ñ*^(*k*)^ is the *k*th cluster mean. The number of clusters *K* is a user-defined parameter.

Subsequent to this clustering, we train a deep-learning model parametrizing the specimen ZLP by extending the approach that we developed in ref (26). The adopted neural network architecture is displayed in Figure 1b, where the inputs are the energy loss *E* and the integrated intensity *N*_tot_. The model parameters θ are determined from the minimization of the cost function
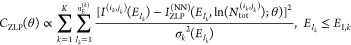
3where within the *k*th thickness cluster a representative spectrum (*i*_*k*_, *j*_*k*_) is randomly selected and with  being the variance within this cluster. The hyperparameters *E*_I,*k*_ in eq 3 define the model training region for each cluster () where the ZLP dominates the total recorded intensity. They are automatically determined from the features of the first derivative d*I*_EELS_/d*E*, e.g., by demanding that only *f*% of the replicas have crossed d*I*_EELS_/d*E* = 0, with *f* ≈ 10%. Typical values of *E*_I,*k*_ are displayed in Figure 1c, where vacuum measurements are also included for reference. To avoid overlearning, the input data is separated into disjoint training and validation subsets, with the latter used to determine the optimal training length using look-back stopping.^24^Figure 1d displays the distribution of the training and validation cost functions, eq 3, evaluated over 5000 models. Both parts c and d of Figure 1 correspond to the WS_2_ nanoflower specimen first presented in ref (26) and revisited here. Further details on the deep-learning model training are reported in section S2.

This procedure is repeated for a large number of models *N*_rep_, each based on a different random selection of cluster representatives, known in this context as “replicas”. One ends up with a Monte Carlo representation of the posterior probability density in the space of ZLP models, providing a faithful estimate of the associated uncertainties
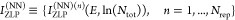
4which makes possible a model-independent subtraction of the ZLP, hence disentangling the contribution from inelastic scatterings *I*_inel_. Following a deconvolution procedure based in discrete Fourier transforms and reviewed in section S3, these subtracted spectra allow us to extract the single-scattering distribution across the specimen and in turn the complex dielectric function from a Kramers–Kronig analysis. In contrast to existing methods, our approach provides a detailed estimate of the uncertainties associated with the ZLP subtraction, and hence quantifies the statistical significance of the determined properties by evaluating confidence level (CL) intervals from the posterior distributions in the space of models.

## Results and Discussion

As a proof of concept we apply our strategy to two different 2D material specimens: First, to horizontally standing WS_2_ flakes belonging to flower-like nanostructures (nanoflowers) characterized by a mixed 2H/3R polytypism. This nanomaterial, a member of the transition metal dichalcogenide (TMD) family, was already considered in the original study^26,27^ and hence provides a suitable benchmark to validate our new strategy. One important property of WS_2_ is that the indirect band gap of its bulk form switches to direct at the monolayer level. Second, to InSe nanosheets prepared by exfoliation of a Sn-doped InSe crystal and deposited onto a holey carbon transmission electron microscopy (TEM) grid. The electronic properties of InSe, such as the band gap value and type, are sensitive to both the layer stacking (β-, γ-, or ε-phase) as well as to the magnitude and type of doping.^28−31^Section S5 provides further details on the structural characterization of the InSe specimen.

**Figure 2:**
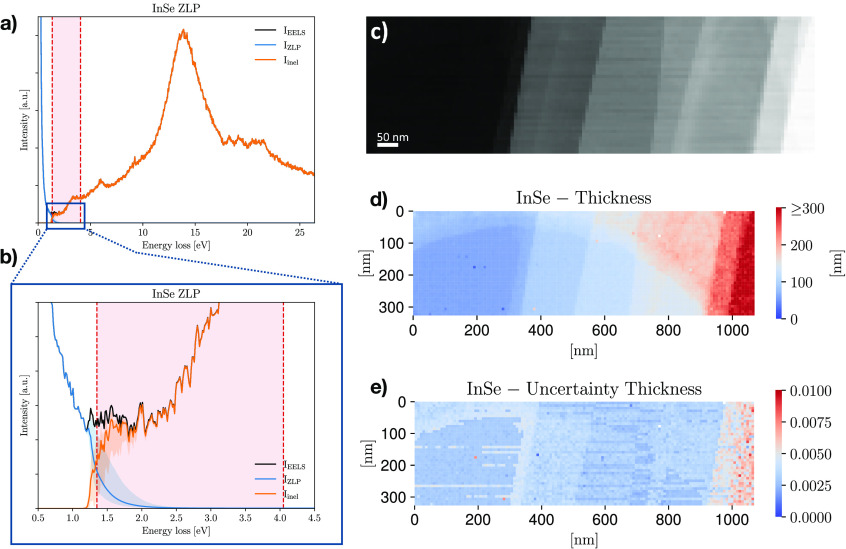
(a) Representative EEL spectrum from the InSe specimen, where we display the data, the ZLP parametrization, and the subtracted inelastic spectrum. The red dashed region indicates the onset of inelastic scatterings where the band gap is extracted. (b) The same spectrum, now zooming in on the low-loss region marked with a blue square in panel a. (c) EELS-SI acquired on the InSe specimen displayed in Figure S5.1, parts a and b, where each pixel corresponds to an individual spectrum. (d and e) The thickness map corresponding to the InSe SI of panel c and the associated relative uncertainties, respectively.

Figure 2a shows a representative EEL spectrum from the InSe specimen, where the original data is compared with the deep-learning ZLP parametrization and the subtracted inelastic contribution. The red dashed region indicates the onset of inelastic scatterings, from which the band gap energy *E*_bg_ and type can be extracted from the procedure described in section S4. In Figure 2b we zoom in on the low-loss region of the same spectrum, where the ZLP and inelastic components become of comparable size. The error bands denote the 68% CL intervals evaluated over *N*_rep_ = 5000 Monte Carlo replicas.

By training the ZLP model on the whole InSe EELS-SI displayed in Figure 2c (see Figure S5.1, parts a and b, for the corresponding STEM measurements) we end up with a faithful parametrization of *I*_ZLP_^(NN)^ (*E*, *N*_tot_) which can be used to disentangle the inelastic contributions across the whole specimen and carry out a spatially resolved determination of relevant physical quantities. To illustrate these capabilities, Figure 2, parts d and e, displays the maps associated with the median thickness and its corresponding uncertainties, respectively, for the same InSe specimen, where a resolution of 8 nm is achieved. One can distinguish the various terraces that compose the specimen, as well as the presence of the hole in the carbon film substrate as a thinner semicircular region; see also the TEM analysis of section S5. The specimen thickness is found to increase from around 20 to up to 300 nm as we move from the left to the right of the map, while that of the carbon substrate is measured to be around 30 nm, consistent with the manufacturer specifications. Uncertainties on the thickness are below the 1% level, as expected since its calculation depends on the bulk (rather than the tails) of the ZLP.

**Figure 3:**
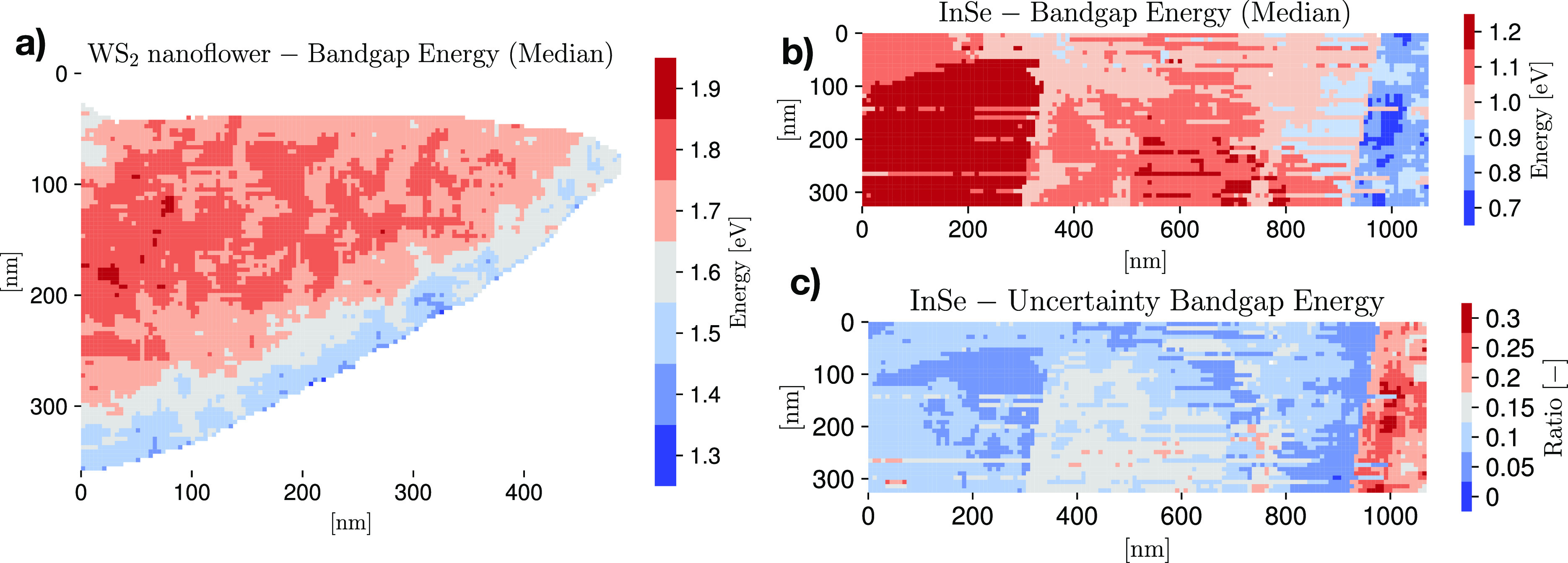
(a) Spatially resolved map of the band gap for the WS_2_ nanoflower specimen, where a mask has been applied to remove the vacuum and pure-substrate pixels. (b and c) The median value of the band gap energy *E*_bg_ and its corresponding 68% CL relative uncertainties across the InSe specimen, respectively.

In the same manner as for the thickness, the ZLP-subtracted SI contains the required information to carry out a spatially resolved determination of the band gap. For this, we adopt the approach of ref (4) where the behavior of *I*_inel_(*E*) in the onset region is modeled as
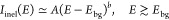
5where both the band gap energy *E*_bg_ and the exponent *b* are extracted from a fit to the subtracted spectra. The value of the exponent is expected to be around *b* ≈ 0.5 (≈1.5) for a semiconductor material characterized by a direct (indirect) band gap. See section S4 for more details of this procedure. Figure 3a displays the band gap map for the WS_2_ nanoflower specimen, where a mask has been applied to remove the vacuum and pure-substrate pixels. A value *b* = 1.5 for the onset exponent is adopted, corresponding to the reported indirect band gap. The uncertainties on *E*_bg_ are found to range between 15% and 25%. The map of Figure 3a is consistent with the findings of ref (26), which obtained a value of the band gap of 2H/3R polytypic WS_2_ of *E*_bg_ = (1.6 ± 0.3) eV with an exponent of *b* = 1.3_–0.7_^+0.3^ from a single spectrum. These results also agree within uncertainties with first-principles calculations based on density functional theory for the band structure of 2H/3R polytypic WS_2_.^32^ Furthermore, the correlation between the thickness and band gap maps points to a possible dependence of the value of *E*_bg_ on the specimen thickness, though this trend is not statistically significant. Further details about the band gap analysis of the WS_2_ nanoflowers are provided in section S6.

Moving to the InSe specimen, parts b and c of Figure 3 display the corresponding maps for the median value of the band gap energy and for its uncertainties, respectively. Photoluminescence (PL) measurements carried out on the same specimen, and described in section S5, indicate a direct band gap with energy value around *E*_bg_ ≈ 1.27 eV; hence, we adopt *b* = 0.5 for the onset exponent. The median values of *E*_bg_ are found to lie in the range between 0.9 and 1.3 eV, with uncertainties of 10–20% except for the thickest region where they are as large as 30%. This spatially resolved determination of the band gap of InSe is consistent with the spatially averaged PL measurements as well as with previous reports in the literature.^33^ Interestingly, there appears to be a dependence of *E*_bg_ with the thickness, with thicker (thinner) regions in the right (left) parts of the specimen favoring lower (higher) values. This correlation, which remains robust once we account for the model uncertainties, is suggestive of the reported dependence of *E*_bg_ in InSe with the number of monolayers.^34^

Within our approach it is also possible to determine simultaneously the exponent *b* together with the band gap energy *E*_bg_. As already observed in ref (26), this exponent is typically affected by large uncertainties. Nevertheless, it is found that in the case of the InSe specimen all pixels in the SI are consistent with *b* = 0.5 and that the alternative scenario with *b* = 1.5 is strongly disfavored. By retaining only those pixels where the determination of *b* is achieved with a precision of better than 50%, one finds an average value of *b* = 0.50 ± 0.26, confirming that indeed this material is a direct semiconductor and in agreement with the spatially integrated PL results. In addition, the extracted values of *E*_bg_ are found to be stable irrespective of whether the exponent *b* is kept fixed or instead is also fitted. Section S8 provides more details on the joint (*E*_bg_, *b*) analysis.

**Figure 4:**
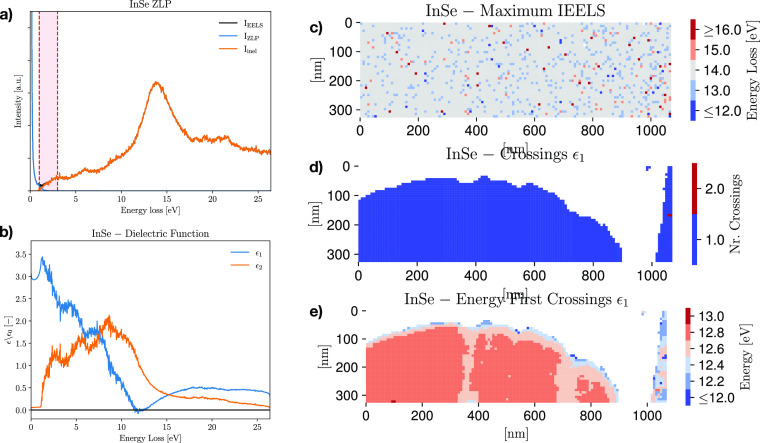
(a) Representative EEL spectrum from the InSe specimen. (b) The real, ϵ_1_(*E*), and imaginary, ϵ_2_(*E*), components of the complex dielectric function associated with the same location. (c) The energy value associated with the global maximum of the inelastic scattering intensity *I*_inel_(*E*) across the InSe specimen. (d and e) The numbers of crossings of ϵ_1_(*E*) and the associated value of the *E*, respectively, across the same specimen, where the SI has been masked to remove pixels with carbon substrate underneath.

We evaluate now the properties of the complex dielectric function ϵ(*E*) using the Kramers–Kronig analysis described in section S3. In the following we focus on the InSe specimen; see section S7 for the corresponding results for the WS_2_ nanoflowers. The local dielectric function provides key information on the nature and location of relevant electronic properties of the specimen. To illustrate the adopted procedure, Figure 4a displays another representative InSe spectrum from the same EELS-SI of Figure 3c. Noticeable features include a marked peak at *E* ≈ 14 eV, corresponding to the bulk plasmon of InSe, as well as a series of smaller peaks in the low-loss region. The real and imaginary parts of the complex dielectric function associated with the same location in the InSe specimen are shown in Figure 4b. The values of the energy loss for which the real component exhibits a crossing, ϵ_1_(*E*_c_) = 0, with a positive slope can be traced back to collective excitations such as a plasmonic resonances. Indeed, one observes how the real component ϵ_1_(*E*) exhibits a crossing in the vicinity of *E* ≈ 13 eV, consistent with the location of the bulk plasmon peak.

Furthermore, the local maxima of the imaginary component ϵ_2_(*E*) can be associated with interband transitions. From Figure 4b, one finds that ϵ_2_(*E*) exhibits local maxima in the low-loss region, immediately after the onset of inelastic scatterings, at energy losses around 3, 6, and 9 eV. The location of these maxima do match with the observed peaks in the low-loss region of Figure 4a, strengthening their interpretation of interband transitions between the valence and conduction bands and consistent also with previous reports in the literature.^35^ The dielectric function in Figure 4b provides also access to ϵ_1_(0), the static dielectric constant, and hence the refractive index *n* of bulk InSe. Our results are in agreement with previous reports^36^ once the thickness of our specimen is taken into account.

As for the thickness and the band gap, one can also map the variation of relevant features in the dielectric function ϵ(*E*) across the specimen. Extending the analysis of parts a and b of Figure 4, Figure 4c shows the value of the energy loss associated with the maximum of the inelastic scattering intensity *I*_inel_(*E*), while parts d and e of Figure 4 display the numbers of crossings of ϵ_1_(*E*) and the corresponding value of the energy loss, respectively. In parts d and e of Figure 4, the SI has been masked to remove pixels with carbon substrate underneath, the reason being that its contribution contaminates the recorded spectra and hence prevents us from robustly extracting ϵ(*E*) associated with InSe. It is found that the specimen exhibits a single crossing whose energy *E*_c_ ranges between 12.5 and 13 eV, close to the maximum of *I*_inel_ and hence consistent with the location of the InSe bulk plasmonic resonance. Uncertainties on *E*_c_ are below the 1% level, since the calculation of ϵ(*E*) depends mildly on the onset region where model errors are the largest. Dielectric function maps such as Figure 4e represent a sensitive method to chart the local electronic properties of a nanostructured material, complementing approaches such as fitting multi-Gaussian models to EEL spectra to identify resonances and transitions. In particular, maps for the local maxima of ϵ_1_(*E*) and ϵ_2_(*E*) could be also be constructed to gauge their variation across the specimen.

Interestingly, as was also the case for the band gap energy in Figure 3c, by comparing Figure 4e with Figure 2d there appears to be a moderate correlation between the crossing energy and the specimen thickness, whereby *E*_c_ decreases as the specimen becomes thicker. While dedicated theoretical and modeling work would be required to ascertain the origin of this sensitivity on the thickness, our results illustrate how our framework makes possible a precise characterization of the local electronic properties of materials at the nanoscale and their correlation with structural features.

## Summary and Outlook

In this work we have presented a novel framework for the automated processing and interpretation of spectral images in electron energy loss spectroscopy. By deploying machine learning algorithms originally developed in particle physics, we achieve the robust subtraction of the ZLP background and hence a mapping of the low-loss region in EEL spectra with precise spatial resolution. In turn, this makes realizing a spatially resolved (≈10 nm) determination of the band gap energy and complex dielectric function in layered materials possible, here represented by 2H/3R polytypic WS_2_ nanoflowers and by InSe flakes. We have also assessed how these electronic properties correlate with structural features, in particular with the local specimen thickness. Our results have been implemented in a new release of the Python open-source EELS analysis framework EELSfitter, available from GitHub (https://github.com/LHCfitNikhef/EELSfitter), together with a detailed online documentation (available from https://lhcfitnikhef.github.io/EELSfitter/index.html).

While here we have focused on the interpretation of EELS-SI for layered materials, our approach is fully general and can be extended both to higher-dimensional data sets, such as momentum-resolved EELS^37^ acquired in the energy-filtered TEM mode, as well as to different classes of nanostructured materials, from topological insulators to complex oxides. One could also foresee extending the method to the interpretation of nanostructured materials stacked in heterostructures and, in particular, to the removal of the substrate contributions, e.g., for specimens fabricated on top of a solid substrate. In addition, in this work we have restricted ourselves to a subset of the important features contained in EEL spectra, while our approach could be extended to the automated identification and characterization across the entire specimen (e.g., in terms of peak position and width) of the full range of plasmonic, excitonic, or intraband transitions to streamline their physical interpretation. Finally, another exciting application of our approach would be to assess the capabilities of novel nanomaterials as prospective light (e.g., sub-GeV) dark matter detectors^38^ by means of their electron energy loss function,^39^ which could potentially extend the sensitivity of ongoing dark matter searches by orders of magnitude.

## Methods

### STEM-EELS Measurements

The STEM-EELS measurements corresponding to the WS_2_ specimen were acquired with a JEOL 2100F microscope with a cold field-emission gun equipped with an aberration corrector operated at 60 kV. A Gatan GIF Quantum ERS system (model 966) was used for the EELS analyses. The spectrometer camera was a Rio (CMOS) camera. The convergence and collection semiangles were 30.0 and 66.7 mrad, respectively. EEL spectra were acquired with an entrance aperture diameter of 5 mm, energy dispersion of 0.025 eV/channel, and exposure time of 0.001 s. For the STEM imaging and EELS analyses, a probe current of 18.1 pA and a camera length of 12 cm were used. The EEL spectrum size in pixels was a height of 94 pixels and a width of 128 pixels. The EELS data corresponding to the InSe specimen were collected in an ARM200F Mono-JEOL microscope equipped with a GIF continuum spectrometer operated at 200 kV. The spectrometer camera was a Rio camera model 1809 (9 megapixels). For these measurements, a slit in the monochromator of 1.3 μm was used. A Gatan GIF Quantum ERS system (model 966) was used for the EELS analyses with convergence and collection semiangles of 23.0 and 21.3 mrad, respectively. EEL spectra were acquired with an entrance aperture diameter of 5 mm, energy dispersion of 0.015 eV/channel, and pixel time of 1.5 s. The EEL spectrum size in pixels was a height of 40 pixels and a width of 131 pixels. For the STEM imaging and EELS analyses, a probe current of 11.2 pA and a camera length of 12 cm were used.

### Photoluminiscence Measurements

The optical spectra were acquired using a home-built spectroscopy setup. The sample was illuminated through a 0.85 NA Zeiss 100× objective. The excitation source was a continuous wave laser with a wavelength of 595 nm and a power of 1.6 mW/mm^2^ (Coherent OBIS LS 594-60). The excitation light was filtered out using color filters (Semrock NF03-594E-25 and FF01-593/LP-25). The sample emission was collected in reflection through the same objective as in excitation and projected onto a CCD camera (Princeton Instruments ProEM 1024BX3) and spectrometer (Princeton Instruments SP2358) via a 4f lens system.

## Supporting Information Available

Technical details about the processing and theoretical interpretation of EEL spectral images and the ZLP subtraction, additional information about the determination of the band gap energy and type as well as of the dielectric function, and details on the structural characterization of the InSe specimen, including PL measurements (PDF)
