# Supplementary material for: Spatially Resolved Band Gap and Dielectric Function in Two-Dimensional Materials from Electron Energy Loss Spectroscopy
Source: J Phys Chem A. 2022 Feb 15;126(7):1255–62. doi: 10.1021/acs.jpca.1c09566 (PMC8883475; doi:10.1021/acs.jpca.1c09566)
Supplement: Supplementary file 1 — jp1c09566_si_001.pdf [file jp1c09566_si_001.pdf]

Supporting Information: Spatially-Resolved Bandgap and Dielectric Function in 2D Materials from Electron Energy Loss Spectroscopy

Abel Brokkelkamp,[†,‖] Jaco ter Hoeve,[‡,¶,‖] Isabel Postmes,[†,‖] Sabrya E. van Heijst,[†] Louis Maduro,[†] Albert V. Davydov,[§] Sergiy Krylyuk,[§] Juan Rojo,[‡,¶] and Sonia Conesa-Boj[*,†]

†*Kavli Institute of Nanoscience, Delft University of Technology, 2628CJ Delft, The Netherlands*

‡*Nikhef Theory Group, Science Park 105, 1098 XG Amsterdam, The Netherlands*

¶*Physics and Astronomy, VU Amsterdam, 1081 HV Amsterdam, The Netherlands*

§*Materials Science and Engineering Division, National Institute of Standards and Technology, Gaithersburg, MD, 20899 USA*

‖*Equal contribution*

E-mail: s.conesaboj@tudelft.nl

In this Supplementary Information we provide technical details to support the results presented in the main manuscript. In particular, we provide information about the processing and theoretical interpretation of EELS Spectral Images, including the ZLP subtraction, about the determination of the bandgap energy and type as well as of the dielectric function, and about the structural characterisation of the InSe specimen.

Contents

S1 Pooling and clustering of EELS-SI S-3

S2 A deep-learning model for the zero-loss peak S-8

S3 Kramers-Kronig analysis of EEL spectra S-12

S4 Band gap analysis of the EELS low-loss region S-22

S5 Structural characterisation of the InSe specimen S-24

S6 Bandgap analysis of 2H/3R WS_2 nanoflowers S-28

S7 Dielectric function in 2H/3R WS_2 nanoflowers S-31

S8 Simultaneous determination of (E_{bg}, b) from EELS-SI S-33

References S-36

S1 Pooling and clustering of EELS-SI

Let us consider a two-dimensional region of the analysed specimen with dimensions $L_x \times L_y$ where EEL spectra are recorded for $n_p = n_x \times n_y$ pixels. Then the information contained within an EELS-SI may be expressed as

$$I^{(i,j)}_{\rm EELS}(E_\ell)\,, \quad i = 1, \ldots, n_x\,, \quad j = 1, \ldots, n_y\,, \quad \ell = 1, \ldots, n_E\,, \tag{S1.1}$$

where $I^{(i,j)}_{\rm EELS}(E_\ell)$ indicates the recorded total electron energy loss intensity for an energy loss E_ℓ for a location in the specimen (pixel) labelled by (i,j), and n_E is the number of bins that compose each spectrum. The spatial resolution of the EELS-SI in the x and y directions is usually taken to be the same, implying that

$$\Delta x = \Delta y \approx \frac{L_x}{n_x} = \frac{L_y}{n_y}\,. \tag{S1.2}$$

For the specimens analysed in this work we have $n_p = \mathcal{O}(10^4)$ spectra corresponding to a spatial resolution of $\Delta x \approx 10$ nm. On the one hand, a higher spatial resolution is important to allow the identification and characterisation of localised features within a nanomaterial, such as structural defects, phase boundaries, surfaces or edges. On the other hand, if the resolution Δx becomes too small the individual spectra become noisy due to limited statistics. Hence, the optimal spatial resolution can be determined from a compromise between these two considerations.

In general it is not known what the optimal spatial resolution should be prior to the STEM-EELS inspection and analysis of a specimen. Therefore, it is convenient to record the spectral image with a high spatial resolution and then, if required, combine subsequently the information on neighbouring pixels by means of a procedure known as pooling or sliding-window averaging. The idea underlying pooling is that one carries out the following replacement for the entries of the EELS spectral image listed in Eq. (S1.1):

$$I^{(i,j)}_{\rm EELS}(E_\ell) \quad \to I^{(i,j)}_{\rm EELS}(E_\ell)\Big|_{\rm pooled} = \frac{1}{N^{(i,j)}_{\rm pool}} \sum_{|i'-i|\le d} \sum_{|j'-j|\le d} \left(\omega_{|i'-i|,|j'-j|} \times I^{(i',j')}_{\rm EELS}(E_\ell) \right)\,, \tag{S1.3}$$

where d indicates the pooling range, $\omega_{|i'-i|,|j'-j|}$ is a weight factor, and the pooling normalisation

is determined by the sum of the relevant weights,

$$N^{(i,j)}_{\rm pool} = \sum_{|i'-i|\le d} \sum_{|j'-j|\le d} \omega_{|i'-i|,|j'-j|} \, . \tag{S1.4}$$

By increasing the pooling range d, one combines the local information from a higher number of spectra and thus reduces statistical fluctuations, at the price of some loss on the spatial resolution of the measurement. For instance, $d = 3/2$ averages the information contained on a 3×3 square centered on the pixel (i, j). Given that there is no unique choice for the pooling parameters, one has to verify that the interpretation of the information contained on the spectral images does not depend sensitively on their value. In this work, we consider uniform weights, $\omega_{|i'-i|,|j'-j|} = 1$, but other options such as Gaussian weights

$$\omega_{|i'-i|,|j'-j|} = \exp\left(-\frac{(i-i')^2}{2d^2} - \frac{(j-j')^2}{2d^2} \right) \, , \tag{S1.5}$$

with $\sigma^2 = d^2$ as variance are straightforward to implement in EELSFitter. The outcome of this procedure is a a modified spectral map with the same structure as Eq. (S1.1) but now with pooled entries. In this work we typically use $d = 3$ to tame statistical fluctuations on the recorded spectra.

As indicated by Eq. (S1.1), the total EELS intensity recorded for each pixel of the SI receives contributions from both inelastic scatterings and from the ZLP, where the latter must be subtracted before one can carry out the theoretical interpretation of the low-loss region measurements. Given that the ZLP arises from elastic scatterings with the atoms of the specimen, and that the likelihood of these scatterings increases with the thickness, its contribution will depend sensitively with the local thickness of the specimen. Hence, before one trains the deep-learning model of the ZLP it is necessary to first group individual spectra as a function of their thickness. In this work this is achieved by means of unsupervised machine learning, specifically with the K-means clustering algorithm. Since the actual calculation of the thickness has as prerequisite the ZLP determination, see Eq. (S3.22), it is suitable to use instead the total integrated intensity as a proxy for the local thickness for the clustering procedure. That is, we cluster spectra as a function of

$$N^{(i,j)}_{\rm tot} \equiv \int_{-\infty}^{\infty} dE \, I^{(i,j)}_{\rm EELS}(E) = \int_{-\infty}^{\infty} dE \, \left(I^{(i,j)}_{\rm ZLP}(E) + I^{(i,j)}_{\rm inel}(E) \right) = N^{(i,j)}_0 + N^{(i,j)}_{\rm inel} \, , \tag{S1.6}$$

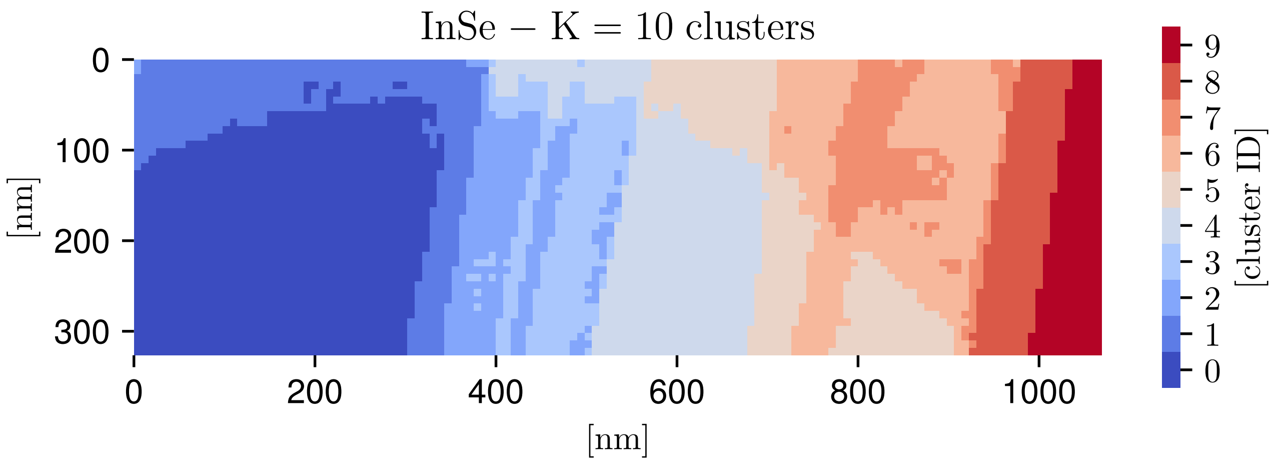

Figure S1.1: The outcome of the K-means clustering procedure applied to the InSe specimen, where each color represents one of the K=10 thickness clusters. This clustering map can be compared with the thickness map of Fig. 2(d), highlighting how the total integrated intensity $N_{\rm tot}$ provides a good proxy for the thickness.

which coincides with the sum of the ZLP and inelastic scattering normalisation factors. Eq. (S1.6) is inversely proportional to the local thickness t and therefore represents a suitable replacement in the clustering algorithm. In practice, the integration in Eq. (S1.6) is restricted to the measured region in energy loss.

The starting point of K-means clustering is a dataset composed by $n_p = n_x \times n_y$ points,

$$\ln\left(N_{\rm tot}^{(r)}\right)\,,\quad r=1,\ldots,n_p\,,\qquad r=i+(n_y-1)j\,, \tag{S1.7}$$

which we want to group into K separate clusters T_k, whose means are given by

$$\ln\left(\widetilde{N}^{(k)}\right)\,,\quad k=1,\ldots,K\,. \tag{S1.8}$$

The cluster means represent the main features of the k-th cluster to which the data points will be assigned in the procedure. Clustering on the logarithm of $N_{\rm tot}^{(r)}$ rather than on its absolute value is found to be more efficient, given that depending on the specimen location the integrated intensity will vary by orders of magnitude.

In K-means clustering, the determination of the cluster means and data point assignments follows from the minimisation of a cost function. This is defined in terms of a distance in specimen

thickness space, given by

$$C_{\rm Kmeans}\left(\boldsymbol{N}_{\rm tot},\boldsymbol{T}\right)=\sum_{r=1}^{n_p}\sum_{k=1}^{K}d_{rk}\left|\ln\left(\frac{\widetilde{N}^{(k)}}{N_{\rm tot}^{(r)}}\right)\right|^p\,, \tag{S1.9}$$

with d_{rk} being a binary assignment variable, equal to 1 if r belongs to cluster k ($d_{rk}=1$ for $r\in T_k$) and zero otherwise, and with the exponent satisfying $p>0$. Here we adopt $p=1/2$, which reduces the weight of eventual outliers in the calculation of the cluster means, and we verify that results are stable if $p=1$ is used instead. Furthermore, since clustering is exclusive, one needs to impose the following sum rule

$$\sum_{k=1}^{K}d_{rk}=1\,,\quad\forall\, r\,. \tag{S1.10}$$

The minimisation of Eq. (S1.9) results in a cluster assignment such that the internal variance is minimised and is carried out by means of a semi-analytical algorithm. This algorithm is iterated until a convergence criterion is achieved, e.g. when the change in the cost function between two iterations is below some threshold. Note that, as opposed to supervised learning, here is it not possible to overfit and eventually one is guaranteed to find the solution that leads to the absolute minimum of the cost function. The end result of the clustering process is that now we can label the information contained in the (pooled) spectral image (for $r=i+(n_y-1)j$) as follows

$$I^{(i,j)}_{{\rm EELS},k}(E_\ell)=\begin{cases}I^{(r)}_{\rm EELS}(E_\ell) & \text{if}\quad r\in T_k\\ 0\quad\text{otherwise}\end{cases}\,,\quad k=1,\ldots,K\,. \tag{S1.11}$$

This cluster assignment makes possible training the ZLP deep-learning model across the complete specimen recorded in the SI accounting for the (potentially large) variations in the local thickness.

The number of clusters K is a free parameter that needs to be fixed taking into consideration how rapidly the local thickness varies within a given specimen. We note that K cannot be too high, else it will not be possible to sample a sufficiently large number of representative spectra from each cluster to construct the prior probability distributions, as required for the Monte Carlo method used in this work. We find that $K=10$ for the InSe and $K=5$ for the WS_2 specimens are suitable choices. Fig. S1.1 displays the outcome of the K-means clustering procedure applied to the InSe specimen, where each color represents one of the K=10 thickness clusters. It can be

compared with the corresponding thickness map in Fig. 2(d); the qualitative agreement further confirms that the total integrated intensity in each pixel $N_{\mathrm{tot}}^{(i,j)}$ represents a suitable proxy for the local specimen thickness.

S2 A deep-learning model for the zero-loss peak

Given that the zero-loss peak background cannot be evaluated from first principles, in this work we deploy supervised machine learning combined with Monte Carlo methods to construct a neural network parameterisation of the ZLP. Within this approach, one can faithfully model the ZLP dependence on both the electron energy loss and on the local specimen thickness. Our approach, first presented in,[S1] is here extended to model the thickness dependence and to the simultaneous interpretation of the $\mathcal{O}(10^4)$ spectra that constitute a typical EELS-SI. One key advantage is the robust estimate of the uncertainties associated to the ZLP modelling and subtraction procedures using the Monte Carlo replica method.[S2]

The neural network architecture adopted in this work is displayed in Fig. 1(b). It contains two input variables, namely the energy loss E and the logarithm of the integrated intensity $\ln(N_{\rm tot})$, the latter providing a proxy for the thickness t. Both E and $\ln(N_{\rm tot})$ are preprocessed and rescaled to lie between 0.1 and 0.9 before given as input to the network. Three hidden layers contain 10, 15, and 5 neurons respectively. The activation state of the output neuron in the last layer, $\xi_1^{(n_L)}$, is then related to the intensity of the ZLP as

$$I_{\rm ZLP}^{(\rm NN)}(E, \ln(N_{\rm tot})) = \exp\left(\xi_1^{(n_L)}(E, \ln(N_{\rm tot}))\right) \,, \tag{S2.1}$$

where an exponential function is chosen to facilitate the learning, given that the EELS intensities in the training dataset can vary by orders of magnitude. Sigmoid activation functions are adopted for all layers except for a ReLU in the final layer, to guarantee a positive-definite output of the network and hence of the predicted intensity.

The training of this neural network model for the ZLP is carried out as follows. Assume that the input SI has been classified into K clusters following the procedure of App. S1. The members of each cluster exhibit a similar value of the local thickness. Then one selects at random a representative spectrum from each cluster,

$$\left\{ I_{\rm EELS}^{(i_1,j_1)}(E), I_{\rm EELS}^{(i_2,j_2)}(E), \ldots, I_{\rm EELS}^{(i_K,j_K)}(E) \right\} \,, \tag{S2.2}$$

each one characterised by a different total integrated intensity evaluated from Eq. (S1.6),

$$\left\{ N_{\rm tot}^{(i_1,j_1)}, N_{\rm tot}^{(i_2,j_2)}, \ldots, N_{\rm tot}^{(i_K,j_K)} \right\} , \tag{S2.3}$$

such that (i_k, j_k) belongs to the k-th cluster. To ensure that the neural network model accounts only for the energy loss E dependence in the region where the ZLP dominates the recorded spectra, we remove from the training dataset those bins with $E \geq E_{{\rm I},k}$ with $E_{{\rm I},k}$ being a model hyperparameter[S1] which varies in each thickness cluster. The cost function $C_{\rm ZLP}$ used to train the NN model is then

$$C_{\rm ZLP} = \frac{1}{n_E} \sum_{k=1}^{K} \sum_{\ell_k=1}^{n_E^{(k)}} \frac{\left[I_{\rm EELS}^{(i_k,j_k)}(E_{\ell_k}) - I_{\rm ZLP}^{(\rm NN)}\left(E_{\ell_k}, \ln\left(N_{\rm tot}^{(i_k,j_k)} \right)\right)\right]^2}{\sigma_k^2\left(E_{\ell_k}\right)} , \qquad E_{\ell_k} \leq E_{{\rm I},k} , \tag{S2.4}$$

where the total number of energy loss bins that enter the calculation is the sum of bins in each individual spectrum, $n_E = \sum_{k=1}^{K} n_E^{(k)}$. The denominator of Eq. (S2.4) is given by $\sigma_k\left(E_{\ell_k}\right)$, which represents the variance within the k-th cluster for a given value of the energy loss E_{ℓ_k}. This variance is evaluated as the size of the 68% confidence level (CL) interval of the intensities associated to the k-th cluster for a given value of E_{ℓ_k}.

For such a random choice of representative cluster spectra, Eq. (S2.2), the parameters (weights and thresholds) of the neural network model are obtained from the minimisation of Eq. (S2.4) until a suitable convergence criterion is achieved. Here this training is carried out using stochastic gradient descent (SGD) as implemented in the `PyTorch` library,[S3] specifically by means of the ADAM minimiser. The optimal training length is determined by means of the look-back cross-validation stopping. In this method, the training data is divided 80%/20% into training and validation subsets, with the best training point given by the absolute minimum of the validation cost function $C_{\rm ZLP}^{(\rm val)}$ evaluated over a sufficiently large number of iterations.

In order to estimate and propagate uncertainties associated to the ZLP parametrisation and subtraction procedure, here we adopt a variant of the Monte Carlo replica method[S1] benefiting from the high statistics (large number of pixels) provided by an EELS-SI. The starting point is selecting $N_{\rm rep} \simeq \mathcal{O}\,(5000)$ subsets of spectra such as the one in Eq. (S2.2) containing one representative of each of the K clusters considered. One denotes this subset of spectra as a Monte Carlo (MC)

replica, and we denote the collection of replicas by

$$\boldsymbol{I}^{(m)} = \left\{ I_{\rm EELS}^{(i_{m,1},j_{m,1})}(E), I_{\rm EELS}^{(i_{m,2},j_{m,2})}(E), \ldots, I_{\rm EELS}^{(i_{m,K},j_{m,K})}(E) \right\} \,, \quad m = 1, \ldots, N_{\rm rep} \,. \tag{S2.5}$$

where now the superindices $(i_{m,k}, j_{m,k})$ indicate a specific spectrum from the k-th cluster that has been assigned to the m-th replica. Given that these replicas are selected at random, they provide a representation of the underlying probability density in the space of EELS spectra, e.g. those spectra closer to the cluster mean will be represented more frequently in the replica distribution.

By training now a separate model to each of the $N_{\rm rep}$ replicas, one ends up with another Monte Carlo representation, now of the probability density in the space of ZLP parametrisations. This is done by replacing the cost function Eq. (S2.4) by

$$C_{\rm ZLP}^{(m)} = \frac{1}{n_E} \sum_{k=1}^{K} \sum_{\ell_k=1}^{n_E^{(k)}} \frac{\left[I_{\rm EELS}^{(i_{m,k},j_{m,k})}(E_{\ell_k}) - I_{\rm ZLP}^{\rm (NN)(m)}\left(E_{\ell_k}, \ln\left(N_{\rm tot}^{(i_{m,k},j_{m,k})} \right) \right) \right]^2}{\sigma_k^2\left(E_{\ell_k}\right)} \,, \qquad E_{\ell_k} \le E_{{\rm I},k} \,, \tag{S2.6}$$

and then performing the model training separately for each individual replica. Note that the denominator of the cost function Eq. (S2.6) is independent of the replica. The resulting Monte Carlo distribution of ZLP models, indicated by

$$\boldsymbol{I}_{\rm ZLP}^{\rm (NN)} = \left\{ I_{\rm ZLP}^{\rm (NN)(1)}\left(E, \ln\left(N_{\rm tot}\right)\right), \ldots, I_{\rm ZLP}^{{\rm (NN)}(N_{\rm rep})}\left(E, \ln\left(N_{\rm tot}\right)\right) \right\} \tag{S2.7}$$

makes possible subtracting the ZLP from the measured EELS spectra following the matching procedure described in[S1] and hence isolating the inelastic contribution in each pixel,

$$I_{\rm inel}^{(i,j)(m)}(E) \simeq \left[I_{\rm EELS}^{(i,j)}(E) - I_{\rm ZLP}^{\rm (NN)(m)}\left(E, \ln\left(N_{\rm tot}^{(i,j)} \right) \right) \right] \,, \quad m = 1, \ldots, N_{\rm rep} \,. \tag{S2.8}$$

The variance of $I_{\rm inel}^{(i,j)}(E)$ over the MC replica sample estimates the uncertainties associated to the ZLP subtraction procedure. By means of these MC samplings of the probability distributions associated to the ZLP and inelastic components of the recorded spectra, one can evaluate the relevant derived quantities with a faithful error estimate. Note that in our approach error propagation is realised without the need to resort to any approximation, e.g. linear error analysis.

One important benefit of Eq. (S2.6) is that the machine learning model training can be carried

out fully in parallel, rather than sequentially, for each replica. Hence our approach is most efficiently implemented when running on a computer cluster with a large number of CPU (or GPU) nodes, since this configuration maximally exploits the parallelization flexibility of the Monte Carlo replica method.

As mentioned above, the cluster-dependent hyperparameters $E_{\mathrm{I},k}$ ensure that the model is trained only in the energy loss data region where ZLP dominates total intensity. This is illustrated by the scheme of Fig. 3.3 in,[S1] which displays a toy simulation of the ZLP and inelastic scattering contributions adding up to the total recorded EELS intensity. The neural network model for the ZLP is then trained on the data corresponding to region I, while region II is obtained entirely from model predictions. To determine the values of $E_{\mathrm{I},k}$, we evaluate the first derivative of the total recording intensity, $dI_{\mathrm{EELS}}(E)/dE$, for each of the members of the k-th cluster. When this derivative crosses zero, the contribution from I_{inel} will already be dominant. There are then two options. First, one sets $E_{\mathrm{I},k} = f \times E_{\mathrm{min},k}$, where $f < 1$ and $E_{\mathrm{min},k}$ is the energy where the median of dI_{EELS}/dE crosses zero (first local minimum) for cluster k. Second, one sets $E_{\mathrm{I},k}$ to be the value where at most $f\%$ of the models have crossed $dI_{\mathrm{EELS}}/dE = 0$, with $f \simeq 10\%$. This choice implies that 90% of the models still exhibit a negative derivative. We have verified that compatible results are obtained with the two choices, indicating that results are reasonably stable with respect to the value of the hyperparameter $E_{\mathrm{I},k}$.

The second model hyperparameter, denoted by $E_{\mathrm{II},k}$ in Fig. 3.3 in,[S1] indicates the region for which the ZLP can be considered as fully negligible. Hence in this region III we impose that $I_{\mathrm{ZLP}}(E) \to 0$ by means of the Lagrange multiplier method. This condition fixes the model behaviour in the large energy loss limit, which otherwise would remain unconstrained. Since the ZLP is known to be a steeply-falling function, $E_{\mathrm{II},k}$ should not chosen not too far from $E_{\mathrm{I},k}$ to avoid an excessive interpolation region. In this work we use $E_{\mathrm{II},k} = 3 \times E_{\mathrm{I},k}$, though this choice can be adjusted by the user.

Finally, we mention that the model hyperparameters $E_{\mathrm{I},k}$ and $E_{\mathrm{II},k}$ could eventually be determined by means of an automated hyper-optimisation procedure as proposed in,[S4] hence further reducing the need for human-specific input in the whole procedure.

S3 Kramers-Kronig analysis of EEL spectra

Here we provide an overview of the theoretical formalism, based on,[S5] adopted to evaluate the single-scattering distribution, local thickness, bandgap energy and type and complex dielectric function from the measured EELS spectra. As indicated by Eq. (1) in the main manuscript, these spectra receive three contributions: the one from inelastic single scatterings off the electrons in the specimen, the one associated to multiple inelastic scatterings, and then the ZLP arising from elastic scatterings and instrumental broadening. Hence a generic EEL spectrum $I_{\rm EELS}(E)$ can be decomposed as

$$I_{\rm EELS}(E) = I_{\rm ZLP}(E) + I_{\rm inel}(E) = I_{\rm ZLP}(E) + \sum_{n=1}^{\infty} I_{\rm inel}^{(n)}(E)\,, \tag{S3.1}$$

where E is the energy loss experienced by the electrons upon traversing the specimen, $I_{\rm ZLP}$ is the ZLP intensity, and $I_{\rm inel}^{(n)}$ indicates the contribution associated to n inelastic scatterings. The ZLP intensity can be further expressed as

$$I_{\rm ZLP}(E) = N_0 R(E)\,, \qquad \int_{-\infty}^{\infty} dE\, R(E) = 1\,, \tag{S3.2}$$

where $R(E)$ is known as the resolution or instrumental response function whose full width at half-maximum (FWHM) indicates the resolution of the instrument. The normalisation factor N_0 thus corresponds to the integrated intensity under the zero-loss peak. In the following, we assume that the ZLP contribution to Eq. (S3.1) has already been disentangled from that associated to inelastic scatterings by means of the subtraction procedure described in App. S2.

The single-scattering distribution. If one denotes by t the local thickness of the specimen and by λ the mean free path of the electrons, then assuming that inelastic scatterings are uncorrelated and that $t \gtrsim \lambda$, one has that the integral over the n-scatterings distribution $I_{\rm inel}^{(n)}$ is a Poisson distribution

$$N_n \equiv \int_{-\infty}^{\infty} dE\, I_{\rm inel}^{(n)}(E) = B \frac{(t/\lambda)^n}{n!} e^{-t/\lambda}\,, \qquad n = 1, 2, \ldots, \infty\,, \tag{S3.3}$$

with B a normalisation constant. From the combination of Eqns. (S3.1) and (S3.3) it follows that

$$N_{\rm inel} \equiv \int_{-\infty}^{\infty} dE\, I_{\rm inel}(E) = \sum_{n=1}^{\infty} N_n = B \sum_{n=1}^{\infty} \frac{(t/\lambda)^n}{n!} e^{-t/\lambda} = B\left(1 - e^{-t/\lambda}\right)\,, \tag{S3.4}$$

and hence one finds that the integral over the n-scatterings distribution is such that

$$N_n = \frac{N_{\rm inel}}{(1-e^{-t/\lambda})}\frac{(t/\lambda)^n}{n!}e^{-t/\lambda}\,, \tag{S3.5}$$

in terms of the normalisation $N_{\rm inel}$ of the full inelastic scattering distribution, the sample thickness t and the mean free path λ. Note also that the ZLP normalisation factor N_0 is then given in terms of the inelastic one as

$$N_0 = \frac{N_{\rm inel}}{e^{t/\lambda}-1}\,, \tag{S3.6}$$

and hence one has the following relations between integrated inelastic scattering intensities

$$\frac{N_1^n}{N_n} = n!N_0^{n-1}\,, \qquad \forall\, n \ge 1\,. \tag{S3.7}$$

In order to evaluate the local thickness of the specimen and the corresponding dielectric function, it is necessary to deconvolute the measured spectra and extract from them the single-scattering distribution (SSD), $I_{\rm SSD}(E)$. The SSD is related to the experimentally measured $n=1$ distribution, $I^{(1)}_{\rm inel}(E)$ by the finite resolution of our measurement apparatus:

$$I^{(1)}_{\rm inel}(E) = R(E)\otimes I_{\rm SSD}(E) \equiv \int_{-\infty}^{\infty} dE'\, R(E-E')I_{\rm SSD}(E')\,, \tag{S3.8}$$

where in the following $\otimes$ denotes the convolution operation. It can be shown, again treating individual scatterings as uncorrelated, that the experimentally measured $n=2$ and $n=3$ multiple scattering distributions can be expressed in terms of the SSD as

$$I^{(2)}_{\rm inel}(E) = R(E)\otimes I_{\rm SSD}(E)\otimes I_{\rm SSD}(E)/\left(2!N_0\right)\,, \tag{S3.9}$$

$$I^{(3)}_{\rm inel}(E) = R(E)\otimes I_{\rm SSD}(E)\otimes I_{\rm SSD}(E)\otimes I_{\rm SSD}(E)/\left(3!N_0^2\right)\,, \tag{S3.10}$$

and likewise for $n \ge 4$. Combining this information, one observes that the spectrum Eq. (S3.1) can be expressed in terms of the resolution function R, the ZLP normalisation N_0, and the single-

scattering distribution $I_{\rm SSD}$ as follows

$$
\begin{aligned}
I_{\rm EELS}(E) &= N_0 R(E) + R(E)\otimes I_{\rm SSD}(E) + R(E)\otimes I_{\rm SSD}(E)\otimes I_{\rm SSD}(E)/\left(2!N_0\right) + \ldots \\
&= R(E)\otimes\left(N_0\delta(E) + I_{\rm SSD}(E) + I_{\rm SSD}(E)\otimes I_{\rm SSD}(E)/\left(2!N_0\right) + \ldots\right) \\
&= N_0 R(E)\otimes\left(\delta(E) + \sum_{n=1}^{\infty}\left[I_{\rm SSD}(E)\otimes\right]^n \delta(E)/\left(n!N_0^n\right)\right)\,, \qquad \text{(S3.11)}
\end{aligned}
$$

where $\delta(E)$ is the Dirac delta function. If the ZLP normalisation factor N_0 and resolution function $R(E)$ are known, then one can use Eq. (S3.11) to extract the SSD from the measured spectra by means of a deconvolution procedure.

SSD deconvolution. The structure of Eq. (S3.11) indicates that transforming to Fourier space will lead to an algebraic equation which can then be solved for the SSD. Here we define the Fourier transform $\widetilde{f}(\nu)$ of a function $f(E)$ as follows

$$
\mathcal{F}\left[f(E)\right](\nu) \equiv \widetilde{f}(\nu) \equiv \int_{-\infty}^{\infty} dE\, f(E) e^{-2\pi i E\nu}\,, \qquad \text{(S3.12)}
$$

whose inverse is given by

$$
\mathcal{F}^{-1}\left[\widetilde{f}(\nu)\right](E) = f(E) \equiv \int_{-\infty}^{\infty} d\nu\, \widetilde{f}(\nu) e^{2\pi i E\nu}\,, \qquad \text{(S3.13)}
$$

which has the useful property that convolutions such as Eq. (S3.8) are transformed into products,

$$
\text{if } f(E) = g(E)\otimes h(E) \quad \text{then} \quad \mathcal{F}\left[f(E)\right] = \widetilde{f}(\nu) = \widetilde{g}(\nu)\widetilde{h}(\nu)\,. \qquad \text{(S3.14)}
$$

The Fourier transform of Eq. (S3.11) leads to the Taylor expansion of the exponential and hence

$$
\widetilde{I}_{\rm EELS}(\nu) = N_0\widetilde{R}(\nu)\exp\left(\frac{\widetilde{I}_{\rm SSD}(\nu)}{N_0}\right)\,, \qquad \text{(S3.15)}
$$

which can be solved for the Fourier transform of the single scattering distribution

$$
\widetilde{I}_{\rm SSD}(\nu) = N_0 \ln\frac{\widetilde{I}_{\rm EELS}(\nu)}{N_0\widetilde{R}(\nu)} = N_0\ln\frac{\mathcal{F}\left[I_{\rm EELS}(E)\right](\nu)}{N_0\mathcal{F}\left[R(E)\right](\nu)}\,. \qquad \text{(S3.16)}
$$

By taking the inverse Fourier transform, one obtains the sought-for expression for the single scattering distribution as a function of the electron energy loss

$$I_{\rm SSD}(E) = N_0 \mathcal{F}^{-1}\left[\ln \frac{\mathcal{F}\left[I_{\rm EELS}\right]}{N_0 \mathcal{F}\left[R\right]} \right] , \tag{S3.17}$$

where the only required inputs are the experimentally measured EELS spectra, Eq. (S3.1), with the corresponding ZLP.

Discrete Fourier transforms. Eq. (S3.17) can be evaluated numerically by approximating the continuous transform Eq. (S3.12) by its discrete Fourier transform equivalent. The discrete Fourier transform of a discretised function $f(E)$ defined at $E_n \in \{E_0, ..., E_{N-1}\}$ is given by:

$$\mathcal{F}_D\left[f(E)\right](\nu_k) = \widetilde{f}(\nu_k) = \sum_{n=0}^{N-1} {\rm e}^{-i2\pi kn/N} f(E_n), \qquad \forall\, k \in \{0, ..., N-1\} , \tag{S3.18}$$

with the corresponding inverse transformation being

$$\mathcal{F}_D^{-1}\left[\widetilde{f}(\nu)\right](E_n) = f(E_n) = \frac{1}{N}\sum_{k=0}^{N-1} {\rm e}^{i2\pi kn/N} \widetilde{f}(\nu_k) \qquad \forall\, n \in \{0, ..., N-1\} . \tag{S3.19}$$

If one approximates the continuous function $f(E)$ by its discretised version $f(E_0 + n\Delta E)$ and likewise $\widetilde{f}(\nu)$ by $\widetilde{f}(k\Delta\nu)$ where $\Delta x \Delta\nu = N^{-1}$ one finds that

$$\widetilde{f}(\nu) \approx \Delta x e^{-i2\pi k\Delta\nu E_0} \mathcal{F}_D\left[f(E)\right] , \tag{S3.20}$$

and likewise for the inverse transform

$$f(E) \approx \frac{1}{\Delta x}\mathcal{F}_D^{-1}\left[\widetilde{g}(k\Delta\nu)\right] , \qquad \widetilde{g}(k\Delta\nu) \equiv e^{i2\pi k\Delta\nu E_0}\widetilde{f}(k\Delta\nu) . \tag{S3.21}$$

In practice, the EELS spectra considered are characterised by a fine spacing in E and the discrete approximation for the Fourier transform produces results very close to the exact one.

Thickness calculation. Once the SSD has been determined by means of the deconvolution procedure summarised by Eq. (S3.17), it can be used as input in order to evaluate the local

sample thickness t from the experimentally measured spectra. Kramers-Kronig analysis provides the following relation between the thickness t, the ZLP normalisation N_0, and the single-scattering distribution,

$$t = \frac{4a_0 F E_0}{N_0\left(1 - \mathrm{Re}\left[1/\epsilon(0)\right]\right)} \int_0^\infty dE \frac{I_{\mathrm{SSD}}(E)}{E \ln\left(1 + \beta^2/\theta_E^2\right)}\,, \tag{S3.22}$$

where we have assumed that the effects of surface scatterings can be neglected. In Eq. (S3.22), $a_0 = 0.0529$ nm is Bohr's radius, F is a relativistic correction factor,

$$F = \frac{1 + E_0/(1022\ \mathrm{keV})}{\left[1 + E_0/(511\ \mathrm{keV})\right]^2}\,, \tag{S3.23}$$

with E_0 being the incident electron energy, $\epsilon(E)$ is the complex dielectric function, and θ_E is the characteristic angle defined by

$$\theta_E = \frac{E}{\gamma m_0 v^2} = \frac{E}{\left(E_0 + m_0 c^2\right)(v/c)^2} \tag{S3.24}$$

with γ being the usual relativistic dilation factor, $\gamma = \left(1 - v^2/c^2\right)^{-1/2}$, and β the collection semi-angle of the microscope.[1] For either an insulator or a semiconductor material with refractive index n, one has that

$$\mathrm{Re}\left[1/\epsilon(0)\right] = n^{-2}\,, \tag{S3.25}$$

while $\mathrm{Re}\left[1/\epsilon(0)\right] = 0$ for a metal or semi-metal. Hence, the determination of the dielectric function is not a pre-requisite to evaluate the specimen thickness, and for given microscope operation conditions we can express Eq. (S3.22) as

$$t = \frac{A}{N_0} \int_0^\infty dE \frac{I_{\mathrm{SSD}}(E)}{E \ln\left(1 + \beta^2/\theta_E^2\right)}\,, \tag{S3.26}$$

with A constant across the specimen. If the thickness of the specimen is already known at some location, then Eq. (S3.26) can be used to calibrate A and evaluate this thickness elsewhere. Furthermore, if the thickness of the material has already been determined by means of an independent experimental technique, then Eq. (S3.22) can be inverted to determine the refractive index n of

[1]Which should not be confused with the normalised velocity often used in relativity, $\beta = v/c$.

an insulator or semi-conducting material using

$$n = \left[1 - \frac{4a_0 F E_0}{N_0 t}\left(\int_0^\infty dE \frac{I_{\rm SSD}(E)}{E\ln\left(1+\beta^2/\theta_E^2\right)}\right)\right]^{-1/2} . \quad \text{(S3.27)}$$

The complex dielectric function. The dielectric function of a material, also known as permittivity, is a measure of how easy or difficult it is to polarise a dielectric material such an insulator upon the application of an external electric field. In the case of oscillating electric fields such as those that constitute electromagnetic radiation, the dielectric response will have both a real and a complex part and will depend on the oscillation frequency ω,

$$\epsilon(\omega) = {\rm Re}\left[\epsilon(\omega)\right] + i{\rm Im}\left[\epsilon(\omega)\right] , \quad \text{(S3.28)}$$

which can also be expressed in terms of the energy $E = \hbar\omega$ of the photons that constitute this electromagnetic radiation,

$$\epsilon(E) = {\rm Re}\left[\epsilon(E)\right] + i{\rm Im}\left[\epsilon(E)\right] . \quad \text{(S3.29)}$$

In the vacuum, the real and imaginary parts of the dielectric function reduce to ${\rm Re}\left[\epsilon(E)\right] = 1$ and ${\rm Im}\left[\epsilon(E)\right] = 0$. Furthermore, the dielectric function is related to the susceptibility χ by

$$\epsilon(E) = 1 - \nu\chi(E) , \quad \text{(S3.30)}$$

where ν is the so-called Coulomb matrix.

The single scattering distribution $I_{\rm SSD}(E)$ is related to the imaginary part of the complex dielectric function $\epsilon(E)$ by means the following relation

$$I_{\rm SSD}(E) = \frac{N_0 t}{\pi a_0 m_0 v^2}{\rm Im}\left[\frac{-1}{\epsilon(E)}\right]\ln\left[1+\left(\frac{\beta}{\theta_E}\right)^2\right] , \quad \text{(S3.31)}$$

in terms of the sample thickness t, the ZLP normalisation N_0, and the microscope operation parameters defined in Sect. S3. We can invert this relation to obtain

$${\rm Im}\left[\frac{-1}{\epsilon(E)}\right] = \frac{\pi a_0 m_0 v^2}{N_0 t}\frac{I_{\rm SSD}(E)}{\ln\left[1+\left(\frac{\beta}{\theta_E}\right)^2\right]} . \quad \text{(S3.32)}$$

Since the prefactor in Eq. (S3.32) does not depend on the energy loss E, we see that $\mathrm{Im}[-1/\epsilon(E)]$ will be proportional to the single scattering distribution $I_{\mathrm{SSD}}(E)$ with a denominator that decreases with the energy (since $\theta_E \propto E$) and hence weights more higher energy losses.

Given that the dielectric response function is causal, the real part of the dielectric function can be obtained from the imaginary one by using a Kramers-Kronig relation of the form

$$\mathrm{Re}\left[\frac{1}{\epsilon(E)}\right] = 1 - \frac{2}{\pi}\mathcal{P}\int_0^\infty dE'\,\mathrm{Im}\left[\frac{-1}{\epsilon(E')}\right]\frac{E'}{E'^2 - E^2}\,, \tag{S3.33}$$

where $\mathcal{P}$ stands for Cauchy's prescription to evaluate the principal part of the integral. A particularly important application of this relation is the $E = 0$ case,

$$\mathrm{Re}\left[\frac{1}{\epsilon(0)}\right] = 1 - \frac{2}{\pi}\mathcal{P}\int_0^\infty dE\,\mathrm{Im}\left[\frac{-1}{\epsilon(E)}\right]\frac{1}{E}\,, \tag{S3.34}$$

which is known as the Kramers-Kronig sum rule. Eq. (S3.34) can be used to determine the overall normalisation of $\mathrm{Im}\left[-1/\epsilon(E)\right]$, since $\mathrm{Re}\left[1/\epsilon(0)\right]$ is known for most materials. For instance, as mentioned in Eq. (S3.25), for an insulator or semiconductor material it is given in terms of its refractive index n.

Once the imaginary part of the dielectric function has been determined from the single-scattering distribution, Eq. (S3.32), then one can obtain the corresponding real part by means of the Kramers-Kronig relation, Eq. (S3.33). Afterwards, the full complex dielectric function can be reconstructed by combining the calculation of the real and imaginary parts, since

$$\epsilon(E) = \mathrm{Re}\left[\epsilon(E)\right] + i\mathrm{Im}\left[\epsilon(E)\right] \equiv \epsilon_1(E) + i\epsilon_2(E)\,, \tag{S3.35}$$

implies that

$$\mathrm{Re}\left[\frac{1}{\epsilon(E)}\right] = \frac{\epsilon_1(E)}{\epsilon_1^2(E) + \epsilon_2^2(E)}\,, \qquad \mathrm{Im}\left[\frac{-1}{\epsilon(E)}\right] = \frac{\epsilon_2(E)}{\epsilon_1^2(E) + \epsilon_2^2(E)}\,, \tag{S3.36}$$

and hence one can express the dielectric function in terms of experimentally accessible quantities,

$$\epsilon(E) = \frac{\mathrm{Re}\left[\frac{1}{\epsilon(E)}\right] + i\mathrm{Im}\left[\frac{-1}{\epsilon(E)}\right]}{\left(\mathrm{Re}\left[\frac{1}{\epsilon(E)}\right]\right)^2 + \left(\mathrm{Im}\left[\frac{-1}{\epsilon(E)}\right]\right)^2}\,. \tag{S3.37}$$

Once the complex dielectric function of a material has been determined, it is possible to evaluate related quantities that also provide information about the opto-electronic properties of a material. One example of this would be the optical absorption coefficient, given by

$$\mu(E) = \frac{E}{\hbar c}\left[2\left(\epsilon_1^2(E)+\epsilon_2^2(E)\right)^{1/2} - 2\epsilon_1(E)\right]^{1/2} , \tag{S3.38}$$

which represents a measure of how far light of a given wavelength $\lambda = hc/E$ can penetrate into a material before it is fully extinguished via absorption processes. Furthermore, combining Eqns. (S3.25) and (S3.36) one has that for a semiconductor material, such as those considered in this work, the refractive index is given by the relation

$$n = \left(\frac{\epsilon_1(0)}{\epsilon_1^2(0)+\epsilon_2^2(0)}\right)^{-1/2} , \tag{S3.39}$$

which implies a positive, non-zero value of the real part of the complex dielectric function at $E = 0$.

The complex dielectric function $\epsilon(E)$ provides direct information on the opto-electronic properties of a material, for example those associated to plasmonic resonances. Specifically, a collective plasmonic excitation should be indicated by the condition that the real part of the dielectric function crosses the x axis, $\epsilon_1(E) = 0$, with a positive slope. These plasmonic excitations typically are also translated by a well-defined peak in the energy loss spectra. Hence, verifying that a plasmonic transition indicated by $\epsilon_1(E) = 0$ corresponds to specific energy-loss features provides a valuable handle to pinpoint the nature of local electronic excitations present in the analysed specimen.

The role of surface scatterings. The previous derivations assume that the specimen is thick enough such that the bulk of the measured energy loss distributions arises from volume inelastic scatterings, while edge- and surface-specific contributions can be neglected. However, for relatively thin samples with thickness t below a few tens of nm, this approximation is not necessarily suitable. Assuming a locally flat specimen with two surfaces, in this case Eq. (S3.1) must be generalised to

$$I_{\rm EELS}(E) = I_{\rm ZLP}(E) + I_{\rm inel}(E) + I_S(E) \tag{S3.40}$$

with $I_S(E)$ representing the contribution from surface-specific inelastic scattering. This surface contribution can be evaluated in terms of the real ϵ_1 and imaginary ϵ_2 components of the complex dielectric function,

$$I_S(E) = \frac{N_0}{\pi a_0 k_0 T}\left[\frac{\tan^{-1}(\beta/\theta_E)}{\theta_E} - \frac{\beta}{\beta^2+\theta_E^2}\right]\left(\frac{4\epsilon_2}{(\epsilon_1+1)^2+\epsilon_2^2} - \mathrm{Im}\left[\frac{-1}{\epsilon(E)}\right]\right) , \tag{S3.41}$$

where the electron kinetic energy is $T = m_e v^2/2$.

The main challenge to evaluate the surface component from Eq. (S3.41) is that it depends on the complex dielectric function $\epsilon(E)$, which in turn is a function of the single scattering distribution obtained from the deconvolution of $I_{\rm inel}(E)$ obtained assuming that $I_S(E)$ vanishes. For not too thin specimens, the best approach is then an iterative procedure, whereby one starts by assuming that $I_S(E) \simeq 0$, evaluates $\epsilon(E)$, and uses it to evaluate a first approximation to $I_S(E)$ using Eq. (S3.41). This approximation is then subtracted from Eq. (S3.40) and hence provides a better estimate of the bulk contribution $I_{\rm inel}(E)$. One can then iterate the procedure until some convergence criterion is met. Whether or not this procedure converges will depend on the specimen under consideration, and specifically on the features of the EELS spectra at low energy losses, $E \lesssim 10$ eV. For the specimens considered in this work, it is found that this iterative procedure to determine the surface contributions converges best provided that the local sample thickness satisfies $t \gtrsim 20$ nm.

Validation. The calculations of the local specimen thickness, Eq. (S3.22), and of the complex dielectric function, Eq. (S3.37) presented in this work have been benchmarked with the corresponding implementation available within the HyperSpy framework.[S6] Provided one inputs the same inelastic spectra and ZLP parametrisation, agreement between the two calculations is obtained. This benchmark is illustrated in Fig. S3.1, which compares the EELSfitter-based results with those available from HyperSpy separately for the real and imaginary components of the dielectric function. Both calculations use for the same input ZLP and inelastic spectra, associated to a representative pixel of the WS_2 nanoflower specimen. Residual differences can be attributed to implementation differences e.g. for the discrete Fourier transforms. This validation test further confirms the robustness of the calculations presented in this work.

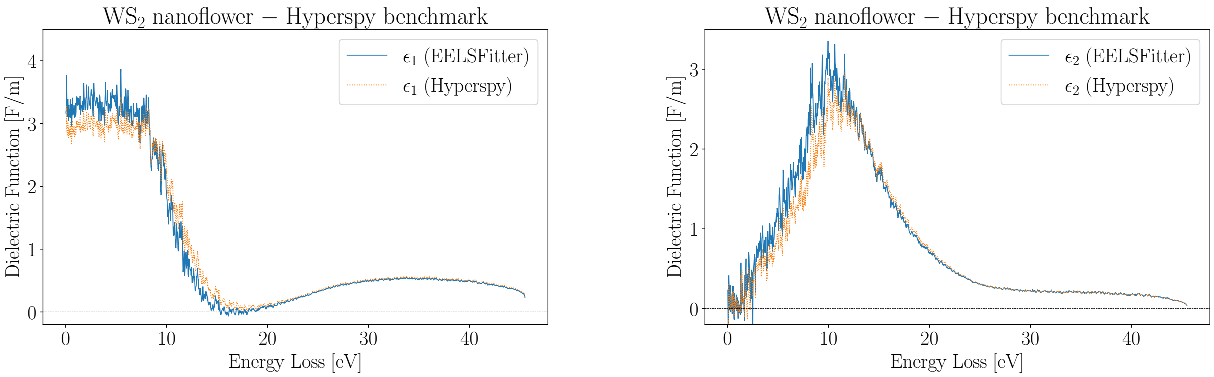

Figure S3.1: Comparison of the EELSFITTER-based results for the real (left) and imaginary (right panel) components of the dielectric function with those available from HYPERSPY for the same input ZLP and inelastic spectrum, associated to a representative pixel of the WS_2 nanoflower specimen.

S4 Band gap analysis of the EELS low-loss region

One important application of ZLP-subtracted EELS spectra is the determination of the bandgap energy and type (direct or indirect) in semiconductor materials. The reason is that the onset of the inelastic scattering intensity provides information on the value of the bandgap energy $E_{\rm bg}$, while its shape for $E \gtrsim E_{\rm bg}$ is determined by the underlying band structure. Different approaches have been put forward to evaluate $E_{\rm bg}$ from subtracted EEL spectra, such as by means of the inflection point of the rising intensity or a linear fit to the maximum positive slope,[S7] see also.[S8] Following,[S1] here we adopt the method of,[S9,S10] where the behaviour of $I_{\rm inel}(E)$ in the region close to the onset of the inelastic scatterings is described by

$$I_{\rm inel}(E) \simeq A\,(E - E_{\rm bg})^{b} \,, \quad E \gtrsim E_{\rm bg} \,, \tag{S4.1}$$

and vanishes for $E < E_{\rm bg}$. Here A is a normalisation constant, while the exponent b provides information on the type of bandgap: it is expected to be $b \simeq 0.5$ (1.5) for a semiconductor material characterised by a direct (indirect) bandgap. While Eq. (S4.1) requires as input the complete inelastic distribution, in practice the onset region is dominated by the single-scattering distribution, since multiple scatterings contribute only at higher energy losses.

The bandgap energy $E_{\rm bg}$, the overall normalisation factor A, and the bandgap exponent b can be determined from a least-squares fit to the experimental data on the ZLP-subtracted spectra. This polynomial fit is carried out in the energy loss region around the bandgap energy, $[E^{\rm (fit)}_{\rm min}, E^{\rm (fit)}_{\rm max}]$. A judicious choice of this interval is necessary to achieve stable results: a too wide energy range will bias the fit by probing regions where Eq. (S4.1) is not necessarily valid, while a too narrow fit range might not contain sufficient information to stabilize the results and be dominated by statistical fluctuation.

Fig. S4.1(a,b) displays representative examples of bandgap fits to the onset of the inelastic spectra in the InSe and WS_2 specimens respectively. The red shaded areas indicate the fitting range, bracketed by $E^{\rm (fit)}_{\rm min}$ and $E^{\rm (fit)}_{\rm max}$. The blue curve and band corresponds to the median and 68% CL intervals of the ZLP-subtracted intensity $I_{\rm inel}(E)$, and the outcome of the bandgap fits based on Eq. (S4.1) is indicated by the green dashed curve (median) and band (68% CL intervals).

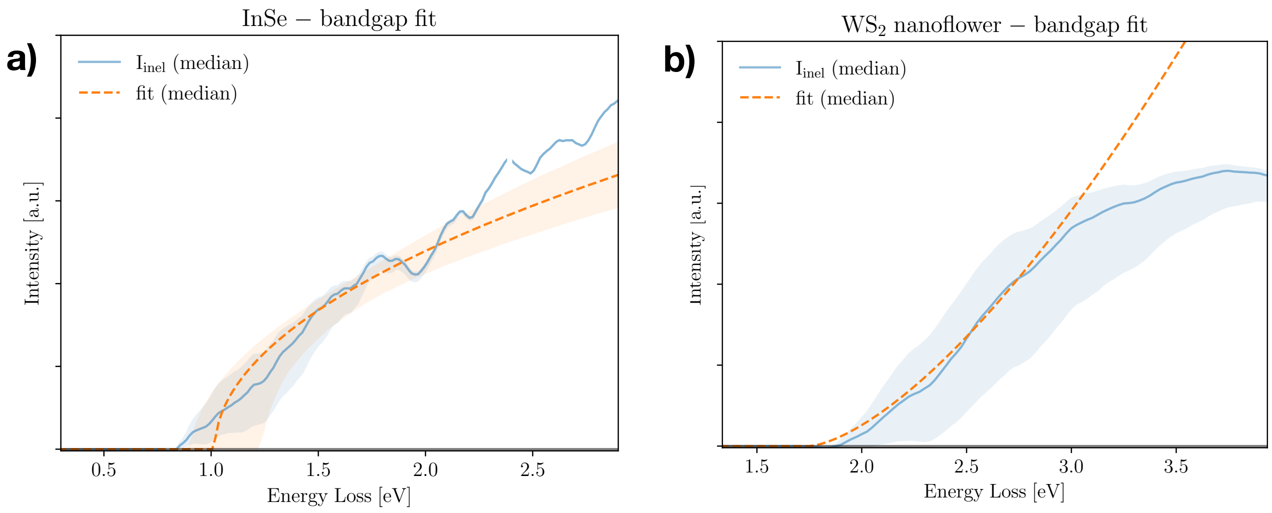

Figure S4.1: Representative examples of bandgap fits to the onset of inelastic spectra in the InSe (a) and WS_2 (b) specimens. The red shaded areas indicate the polynomial fitting range, the blue curve and band corresponds to the median and 68% CL intervals of the ZLP-subtracted intensity $I_{\rm inel}(E)$, and the outcome of the bandgap fits based on Eq. (S4.1) is indicated by the orange dashed curve (median) and band (68% CL intervals).

Here the onset exponents b have been kept fixed to $b = 0.5$ (1.5) for the InSe (WS_2) specimen given the direct (indirect) nature of the underlying band-gaps. One observes how the fitted model describes well the behaviour of $I_{\rm inel}(E)$ in the onset region for both specimens, further confirming the reliability of our strategy to determine the bandgap energy $E_{\rm bg}$. As mentioned in,[S10] it is important to avoid taking a too large interval for $[E^{\rm (fit)}_{\rm min}, E^{\rm (fit)}_{\rm max}]$, else the polynomial approximation ceases to be valid, as one can also see directly from these plots.

S5 Structural characterisation of the InSe specimen

Here we provide details on the structural characterisation of the n-doped InSe specimens. Each specimen is composed by a InSe nanosheet exhibiting a range of thicknesses. The electronic properties of InSe, such as the band gap value and type, are sensitive to both the layer stacking (β, γ, or ε-phase) as well as to the magnitude and type of doping.[S11–S14] In particular, n-doped ε-phase InSe has been reported to exhibit a direct bandgap with value $E_{\mathrm{bg}} = 1.25$ eV.[S15]

These InSe specimens have been grown by means of the Bridgman-Stockbarger method. Doping with Sn impurities is used to obtain n-type InSe. InSe flakes are obtained from bulk material by the sonication procedure,[S16] whereby single InSe crystals are pulverized and added to IPA with a ratio of 2:1 (mg:ml). This combination is then sonicated in a sonic bath for 6 hours while keeping the temperature in the range between 25 °C and 35 °C. The ultra high frequencies lead to gas formation between the layers of the material, building up pressure until adjacent layers split apart. The flakes in the resulting suspension are then collected and dispersed on top of a TEM grid by pipetting.

Fig. S5.1(a) displays a low-magnification High-Angle Annular Dark Field (HAADF) STEM image of one of the specimens obtained from this procedure. The flake is lying on top of the holey carbon grid, and most of its volume is on top of the vacuum. Fig. S5.1(b) shows then a magnification of the region indicated with a red square in (a), while in turn the red rectangle in (b) marks the region where the corresponding EELS-SI, provided in Fig. S5.1(c), has been extracted. The spatial resolution in this EELS-SI is around 8 nm. Note that the (artificial) grey-scale convention adopted is different in Figs. S5.1(b) and (c). It can be observed that most of the flake turns out to be bulk, exhibiting thicknesses of several monolayers at least, with some thinner regions at the edges.

In order to identify the crystalline phase of the specimen under consideration, Fig. S5.2(a) displays a low-magnification HAADF-STEM image of a different InSe flake. This flake has been obtained from the same bulk material as that of Fig. S5.1(a) and hence shares its crystalline structure. Notice how this InSe flake is standing on top of a hole of the TEM grid. Fig. S5.2(b) then shows a high-resolution HAADF-STEM image corresponding to the red square in (a), and whose inset highlights the atomic arrangement in the region indicated with a red square. Fig. S5.2(c)

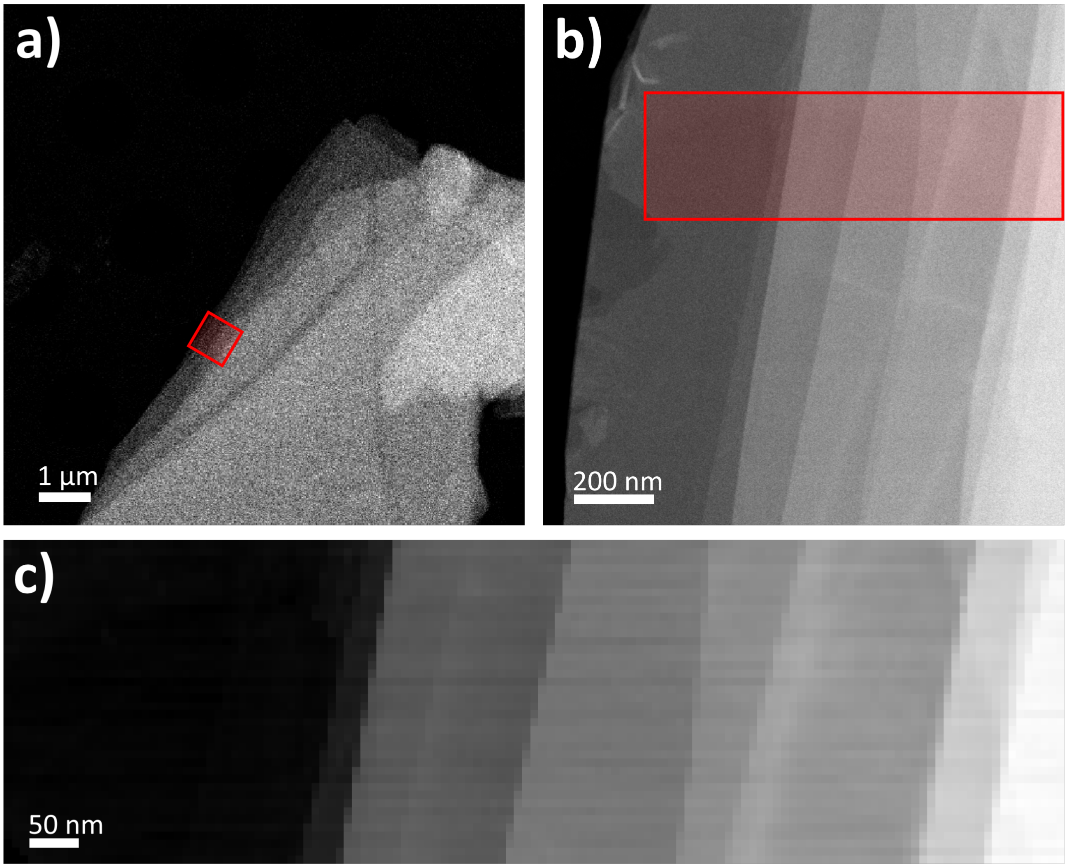

Figure S5.1: (a) Low-magnification HAADF-STEM image of one of the n-doped InSe specimens analysed in this work. (b) Magnification of the region indicated with a red square in (a). (c) EELS-SI acquired on the region indicated with a red rectangle in (b). Note that the grey-scale is different in each panel.

provides the HAADF intensity line profile taken along the blue rectangle in (b), where the three colors correspond to the three-fold periodicity observed in the line profile. HAADF-STEM images are approximately proportional to $Z^{1.7}$, with Z being the atomic number. By comparing with the expectations based on possible atomic models, these images provide useful information to identify the underlying crystalline sequence. The line profile of Fig. S5.2(c) is consistent with the atomic model of ε-phase InSe shown in Fig. S5.2(d), both for top-view and for cross-view, and which uses the same choice of colors as in Fig. S5.2(c).

The structural analysis presented in Fig. S5.2 indicates that the n-doped InSe specimens considered in this work exhibit a crystalline structure characterised by a pure ε-phase. In order to further elucidate the type of the bandgap exhibited by this material, photoluminescence (PL) measurements are carried out. The results, displayed in Fig. S5.3, exhibit a well-defined peak located around 1.26 eV. Hence, we conclude that this material is characterised by a direct bandgap with

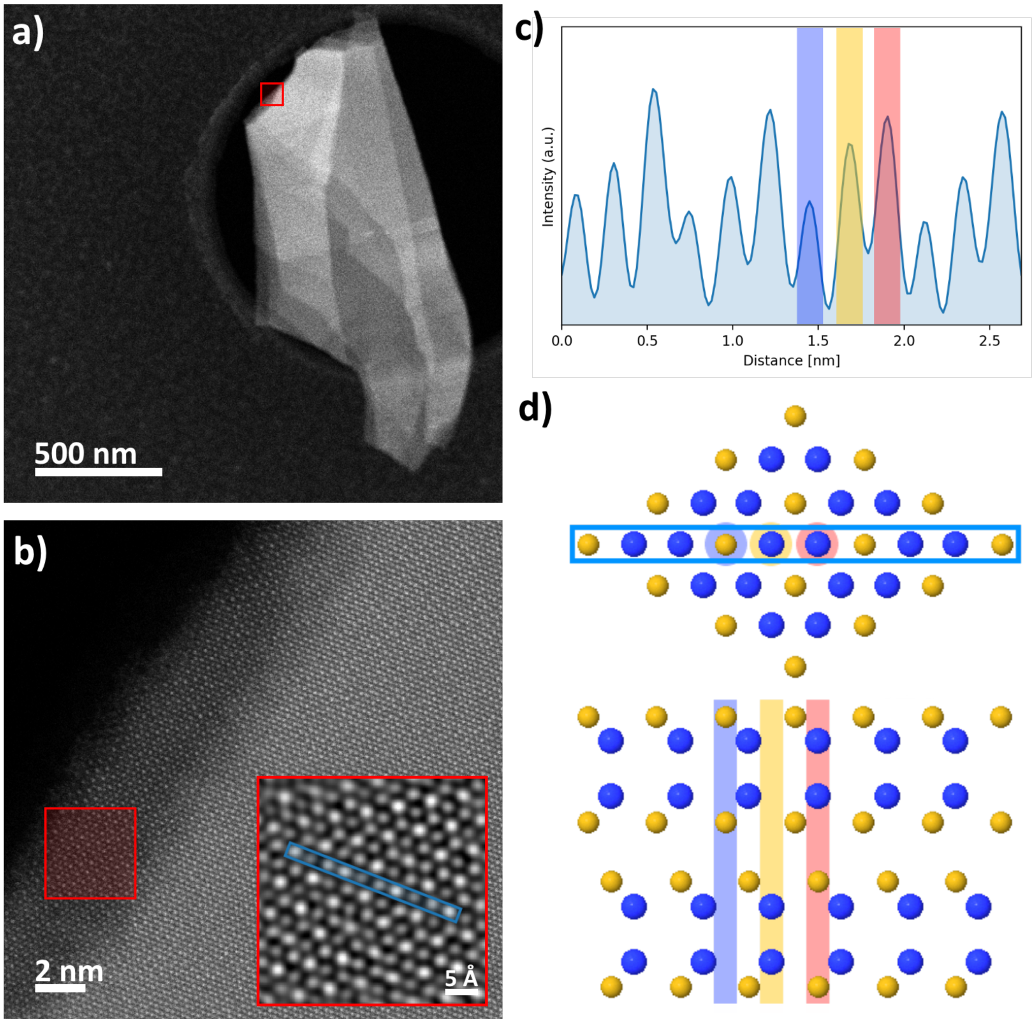

Figure S5.2: (a) Low-magnification HAADF-STEM image of another n-doped InSe flake standing on top of a hole in the TEM grid. (b) High-resolution HAADF-STEM image acquired in the red square in (a), where the inset highlights the atomic crystalline structure. (c) HAADF intensity line profile taken along the blue rectangle in (b), where the three colors correspond to the three-fold periodicity observed in the line profile. (d) Atomic model of ε-phase InSe, with the same choice of colors labeling the atomic layers as in (c), displaying top-view (upper) and cross-view (lower panel).

energy value $E_{\mathrm{bg}} \approx 1.26$ eV, consistent with the findings of.[S15] Note that PL measurements are characterised by a limited spatial resolution as compared to the STEM-EELS results, and therefore this bandgap value corresponds to an average across the specimen. Hence, PL results are not sensitive to spatially-resolved features in the bandgap map such as those reported in Fig. 3(b) of the main manuscript.

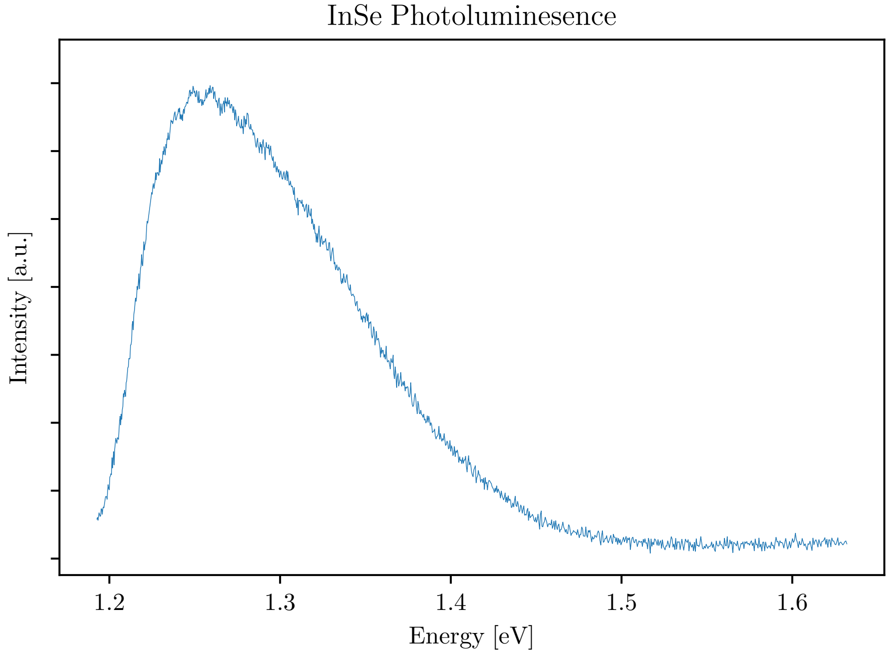

Figure S5.3: Photoluminescence spectrum acquired in the n-doped InSe specimen. A well-defined peak, is observed indicating that this material is characterised by a direct bandgap with energy value $E_{\mathrm{bg}} \approx 1.26$ eV.

S6 Bandgap analysis of 2H/3R WS_2 nanoflowers

Here we apply our new approach to the bandgap analysis of same WS_2 specimen considered in the original study.[S1,S17] This specimen consisted on a horizontally-standing WS_2 flake belonging to flower-like nanostructures characterised by a mixed 2H/3R polytypism. While[S1] restricted its bandgap analysis to a small subset of individual EELS spectra, here we extend it to the whole specimen and as a byproduct also we provide the local thickness map. The goal is to demonstrate how our updated analysis is consistent with the results presented in.[S1] The corresponding results for the dielectric function are presented in App. S7.

Fig. S6.1 displays the outcome of the thickness map determination obtained using the EELS-SI from Fig. 5.1(a) of[S1] as input. Fig. S6.1(a) shows the results of the K-means clustering procedure for $K = 5$. The choice of $K = 5$ clusters is found to be a good compromise between minimizing the variance within each cluster while ensuring a sufficiently large number of members, as required by the applicability the Monte Carlo replica method. The WS_2 specimen itself turns out to be classified into three thickness clusters, surrounded by vacuum (dark blue) at the top and by the SiN substrate (red) in the bottom-right region of the map. Then Fig. S6.1(b) displays the corresponding thickness map evaluated by means of Eq. (S3.22). Note that the image has been masked by retaining only the pixels associated to the WS_2 specimen, to ease visualization. The qualitative agreement with the outcome of the K-means clustering confirms the reliability of the total integrated intensity N_{tot} as a suitable proxy for the local specimen thickness when modelling the ZLP parametrisation.

Fig. S6.2 then presents the corresponding bandgap analysis obtained from the same EELS-SI used to evaluate Fig. S6.1, where again the maps have been filtered such that only those pixels corresponding to the WS_2 specimen are retained. First of all, Fig. S6.2(a) displays the spatially-resolved map displaying the median value of the bandgap energy E_{bg} evaluated across the WS_2 specimen, where the spatial resolution achieved is around 10 nm. These bandgap energies have been obtained from the procedure described in App. S4, specifically by fitting Eq. (S4.1) to the onset of the inelastic spectra. A fixed value of the exponent $b = 1.5$, corresponding to the indirect bandgap reported for this material, is used to stabilize the model fit. Then Fig. S6.2(b) shows the associated relative uncertainty δE_{bg} on the extracted bandgap energy. It is estimated as half

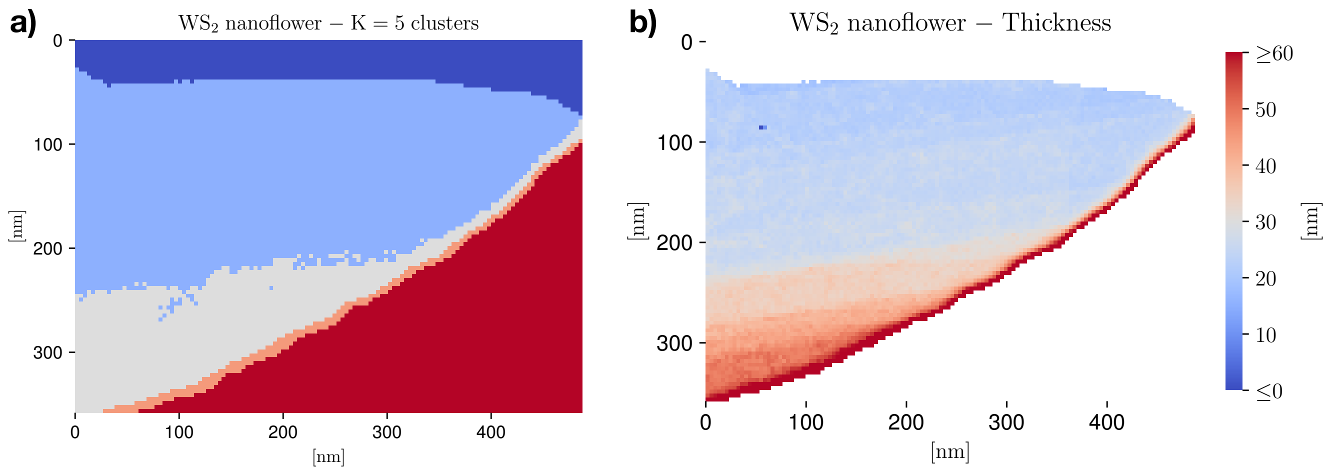

Figure S6.1: Thickness analysis obtained from the EELS-SI presented in Fig. 5.1(a) of[S1] as input. The specimen under consideration is a horizontally-standing flake belonging to a 2H/3R polytypic WS_2 flower-like nanostructure. (a) The outcome of the K-means clustering procedure for $K = 5$. (b) The resulting thickness map. Only pixels associated to the WS_2 specimen are displayed.

the magnitude of the 68% CL interval (corresponding to one standard deviation for a Gaussian distribution) from the Monte Carlo replica sample for each pixel of the SI. One finds that the typical uncertainties $\delta E_{\rm bg}$ range between 15% and 25%. Finally, Fig. S6.2(c) indicates the lower limit of the 68% CL interval for $E_{\rm bg}$.

Ref.[S1] reported a value of the bandgap of 2H/3R polytypic WS_2 of $E_{\rm bg} = (1.6 \pm 0.3)$ eV with a exponent of $b = 1.3^{+0.3}_{-0.7}$ extracted from single EELS spectrum. From the spatially-resolved bandgap maps of Fig. S6.2, one observes how our updated results are in agreement with those from the previous study within uncertainties. Furthermore, this spatially-resolved determination of $E_{\rm bg}$ is in agreement within uncertainties with first-principles calculations based on Density Functional Theory (DFT) of the band structure of 2H/3R polytypic WS_2.[S18] These DFT calculations, which also account for spin-orbit coupling effects, find values of $E_{\rm bg}$ in the range between 1.40 eV and 1.48 eV depending on the settings of the calculation. The DFT predictions are hence consistent with the 68% CL interval for the $E_{\rm bg}$ for a wide region of the specimen, as indicated by Fig. S6.2(c).

Furthermore, inspection of the thickness and bandgap maps, Figs. S6.1(b) and S6.2(a) respectively, reveals an apparent dependence of the value of $E_{\rm bg}$ on the local specimen thickness. Specifically, the bandgap energies tend to increase in the thinner region of the specimen, with $t \approx 25$ nm, and then to decrease as one moves towards the thicker regions with $t \approx 50$ nm. While this dependence with the thickness is suggestive of the known property of WS_2 that $E_{\rm bg}$ increases

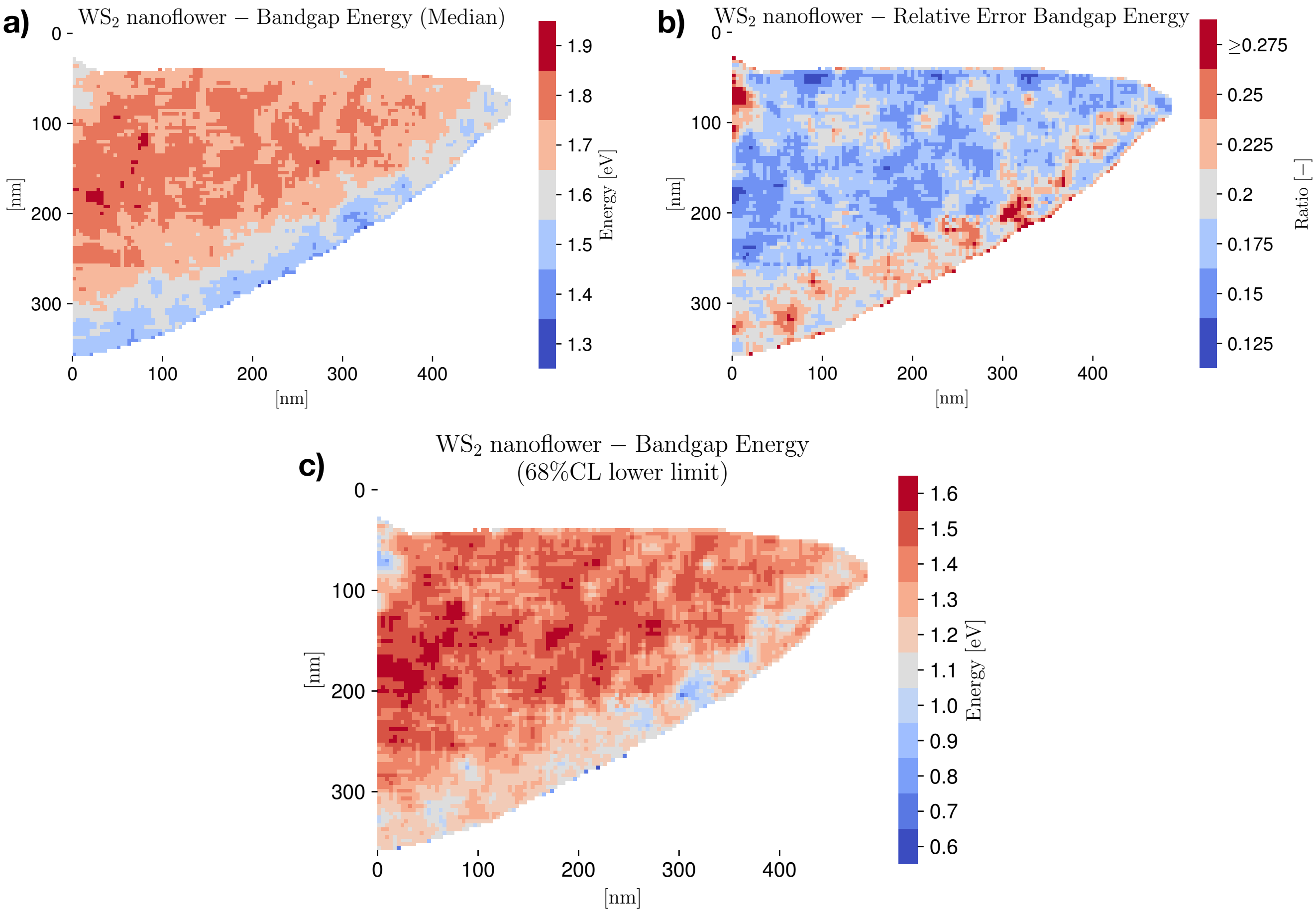

Figure S6.2: Bandgap analysis of the same EELS-SI used to evaluate Fig. S6.1. (a) Spatially-resolved map displaying the median value of the bandgap energy $E_{\rm bg}$ evaluated across the specimen. (b) Same as (a) for the corresponding relative uncertainty $\delta E_{\rm bg}$, evaluated as half of the 68% CL interval from the Monte Carlo replica sample. (c) Same as (a) indicating the lower limit of the 68% CL interval for $E_{\rm bg}$. In the three panels, the maps have been filtered such that only those pixels corresponding to the WS_2 specimen are retained.

when going from bulk to monolayer form, uncertainties remain too large to be able to assign significance to this effect.

S7 Dielectric function in 2H/3R WS_2 nanoflowers

App. S6 characterizes the local thickness and the bandgap energy of the 2H/3R WS_2 nanoflower specimen from[S1,S17] across the whole EELS-SI. We now present the corresponding results for the spatially-resolved determination of the real, $\epsilon_1(E)$, and imaginary, $\epsilon_2(E)$, parts of its complex dielectric function. Fig. S7.1 displays $\epsilon_1(E)$ and $\epsilon_2(E)$ corresponding to two representative spectra of this WS_2 nanoflower specimen. In this analysis we account for the effects of the surface contributions and the error bands quantify the uncertainties associated to the ZLP subtraction procedure.

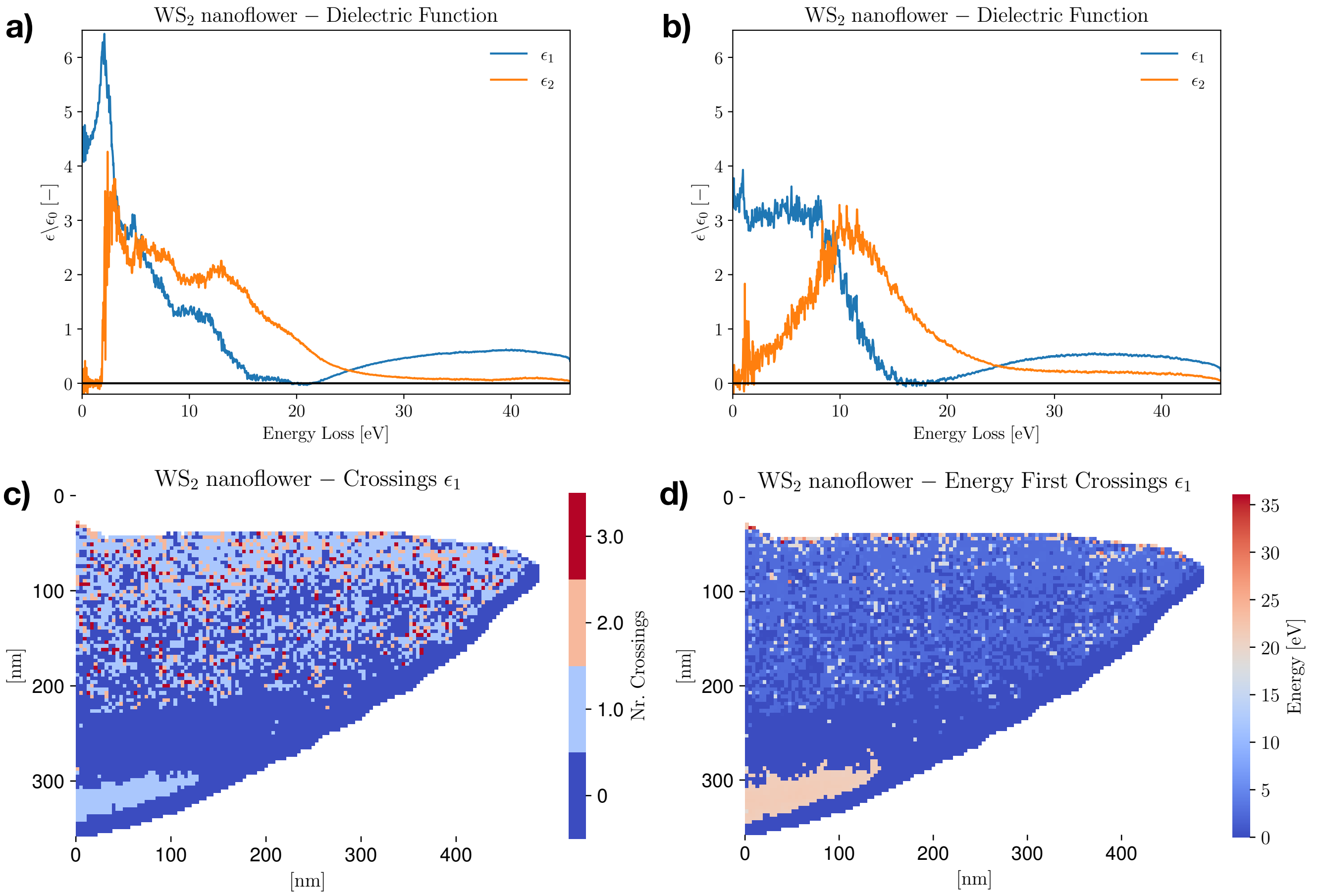

Figure S7.1: (a,b) The real $\epsilon_1(E)$ and imaginary $\epsilon_2(E)$ components of the complex dielectric function corresponding to two representative spectra of the WS_2 nanoflower specimen. The energy values for which the real component exhibits a crossing, $\epsilon_1(E) = 0$ with positive slope, can be attributed to collective electronic transitions. (c) The number of crossings exhibited by the real part of the dielectric function for the same specimen. (d) The energy value of the left-most crossing in (c) for the pixels with ≥ 1 crossings. Both in (c) and (d) only the pixels corresponding to the specimen are retained.

Of particular interest are the values of the energy loss for which the real component of the

dielectric function exhibits a crossing, $\epsilon_1(E) = 0$ with a positive slope. These crossings can be interpreted as indicating a phase transition involving a collective electronic excitation, such as a plasmonic resonance. Here we define that a crossing takes place wherever $\epsilon_1(E) = 0$ at the 90% CL as estimated from the Monte Carlo representation. For the selected spectra displayed in Fig. S7.1(a,b), this condition is satisfied for $E \simeq 22$ eV and $E \simeq 18$ eV respectively. These values are consistent with the bulk and surface plasmonic resonances in 2H/3R polytypic WS_2 identified in.[S17]

Fig. S7.1(c) displays the number of such crossings exhibited by the real part of the dielectric function across the whole WS_2 nanoflower specimen. The vacuum and substrate regions have been masked such that only the spectra corresponding to the specimen are retained. One finds that the majority of the spectra are characterised by either one or zero crossings, while a minority showing two or even three crossings. By comparing with Fig. S6.1(b), one observes how it is in the thicker region of the specimen for which the condition $\epsilon_1(E) = 0$ is typically not satisfied, with the exception of the very bottom region which consistently displays one crossing.

Finally, Fig. S7.1(d) displays the energy E associated to the left-most crossing in Fig. S7.1(c) for those pixels with ≥ 1 crossings. For the upper region of the specimen (characterised by smaller thicknesses), the first crossing is found to be in the low-loss region, while in the bottom (thicker) region, one has a first crossing at $E_c \simeq 21$ eV consistent with the WS_2 bulk plasmon peak. We note that one could also show the values of E_c for the subsequent crossings, for those pixels exhibiting more than one crossing.

S8 Simultaneous determination of $(E_{\rm bg}, b)$ from EELS-SI

As discussed in the SI Sect. S4, the ZLP-subtracted EELS spectra can be used to extract the value of the bandgap energy $E_{\rm bg}$ as well as its type (direct or indirect) in semiconductor material by fitting a functional form

$$I_{\rm inel}(E) \simeq A\,(E - E_{\rm bg})^b\ , \quad E \gtrsim E_{\rm bg}\ , \tag{S8.1}$$

to the onset region of the subtracted inelastic spectra, where $b = 0.5$ (1.5) corresponds to a direct (indirect) semiconductor. In the results presented in the main manuscript, in particular in Fig. 3, fits based on Eq. (S8.1) were carried out by fixing the value of the exponent b to $b = 0.5$ for InSe specimen, motivated by the PL analysis, and $b = 1.5$ for the WS_2 nanoflower, justified by previous work[S1,S17] as well as by independent first-principles DFT calculations.[S18] Here we present results of fits were both the bandgap energy $E_{\rm bg}$ and type b are simultaneously determined from the ZLP-subtracted EELS spectra in the case of the InSe specimen.

Already in our original study,[S1] it was demonstrated that our method is suitable for the joint extraction of both the bandgap energy $E_{\rm bg}$ and the exponent b simultaneously, but that in general the latter is affected by sizable uncertainties. For instance, for two of the EEL spectra considered there from the WS_2 specimen, we found values $b = 1.3^{+0.3}_{-0.7}$ and $b = 1.3^{+0.3}_{-0.4}$, consistent with the theoretical expectations for an indirect semiconductor. One option to reduce the model uncertainties on the exponent parameter b would be to increase the pooling degree of the pixels in the SI, which would reduce the fluctuations in the low-loss region at the price of a degradation of the achieved spatial resolution. Here instead we show how we can obtain important information about the values of the exponent b in a fully data-driven manner without compromising the spatial resolution. The strategy is to mask away the pixels where the joint determination of $(E_{\rm bg}, b)$ is too noisy, and hence retain only those pixels where the relative uncertainties on the two parameters is below a precision threshold.

Figs. S8.1 and S8.2 display the outcome of joint fits of $(E_{\rm bg}, b)$ where these two parameters are extracted simultaneously from the low-loss region of the subtracted EELS spectra for the InSe specimen. The upper panels display the median value and 68% CL relative uncertainty while the bottom panels show the values of the upper and lower ranges of the 68% CL interval, in the case of the exponent b and of the bandgap energy $E_{\rm bg}$ respectively. Only those pixels where the relative

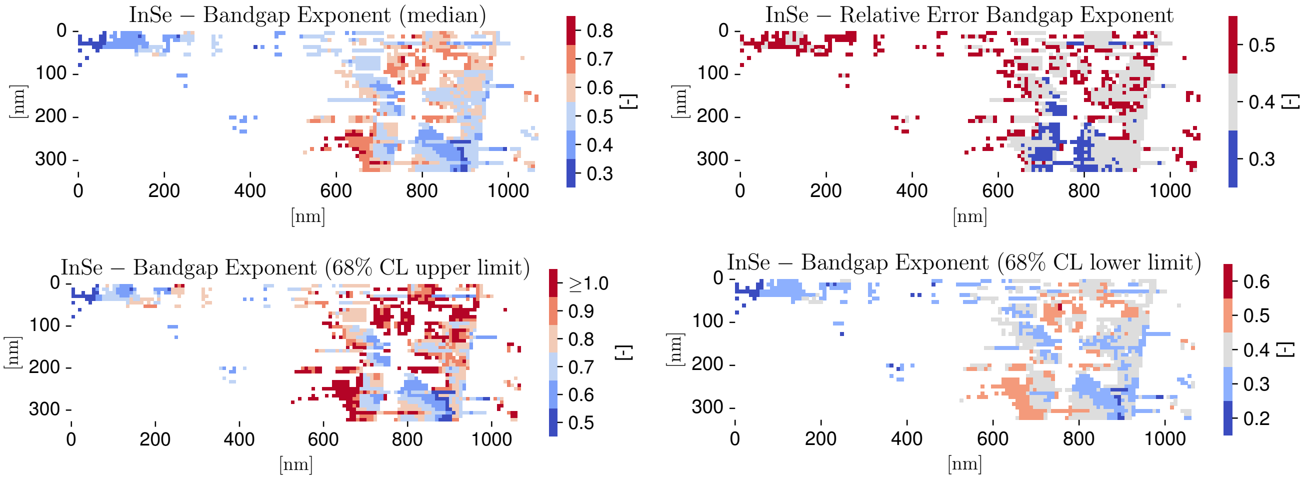

Figure S8.1: Upper panels: the median value (left) and 68% CL relative uncertainty (right panel) for the exponent b in joint fits where the values of $(E_{\rm bg}, b)$ are extracted simultaneously from the low-loss region of the subtracted EELS spectra corresponding to the InSe specimen. Only those pixels where the relative uncertainty in the determination of both b and $E_{\rm bg}$ is below the 50% level are retained. Bottom panels: same, now corresponding to the upper (left) and lower (right panel) ranges of the 68% CL interval for b.

uncertainty in the determination of both b and $E_{\rm bg}$ is below the 50% level are retained, and the rest are masked away. One finds that for around 25% of the pixels it is possible to achieve a reasonable precision in these joint fits of $(E_{\rm bg}, b)$.

Inspection of Figs. S8.1 and S8.2 reveals that the EELS data strongly prefers a value of the exponent around $b \simeq 0.5$, consistent with both the PL measurements and with the expectations of this material being a direct semiconductor, while an alternative scenario with $b = 1.5$ is strongly disfavored. Furthermore, one can verify that for all other pixels in the SI, including the ones masked away, $b = 0.5$ is contained within the 68% CL uncertainty range. If we average over all the SI pixels displayed in Fig. S8.1 we find $b = 0.50 \pm 0.26$, confirming that indeed the specimen is a direct semiconductor. In addition, by comparing with Fig. 3 (b,c) of the main manuscript, one finds that the extracted values of $E_{\rm bg}$ are stable irrespectively of whether the exponent b is kept fixed or instead is fitted.

The analysis presented here demonstrates that our method is also suitable to provide spatially-resolved information about the value of the bandgap type, confirming the value of b obtained from the spatially-averaged PL data. Further work will investigate how to improve the trade-off between spatial resolution and precision in the joint fits of $(E_{\rm bg}, b)$ from the subtracted EELS spectra.

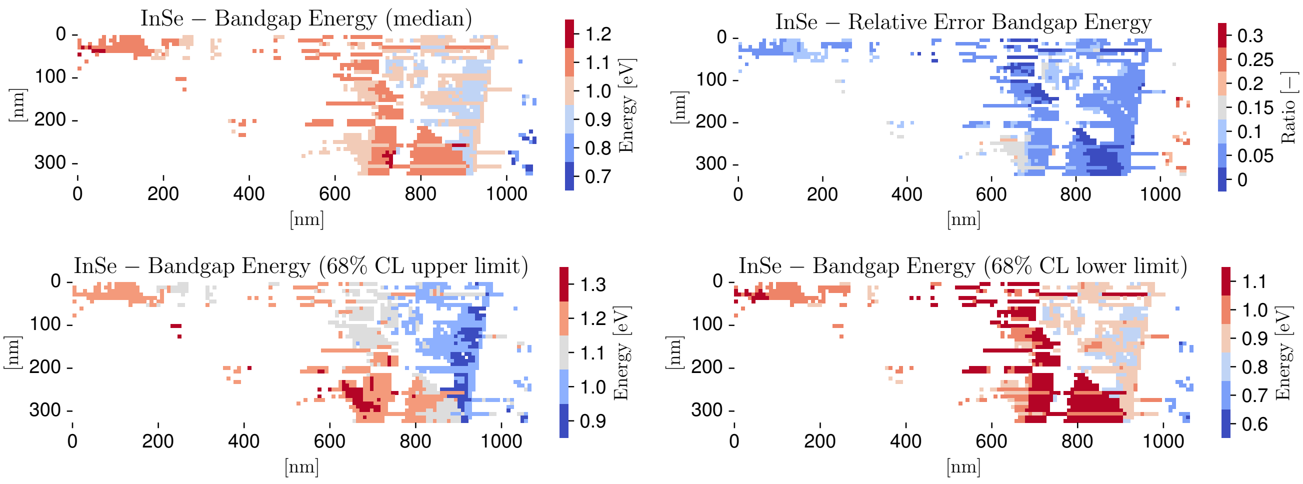

Figure S8.2: Same as Fig. S8.1 now displaying the corresponding results for the bandgap energy E_{bg}.

References

(S1) Roest, L. I.; van Heijst, S. E.; Maduro, L.; Rojo, J.; Conesa-Boj, S. Charting the low-loss region in Electron Energy Loss Spectroscopy with machine learning. *Ultramicroscopy* **2021**, *222*, 113202.

(S2) Del Debbio, L.; Forte, S.; Latorre, J. I.; Piccione, A.; Rojo, J. Neural network determination of parton distributions: The nonsinglet case. *JHEP* **2007**, *03*, 39.

(S3) Paszke, A.; Gross, S.; Massa, F.; Lerer, A.; Bradbury, J.; Chanan, G.; Killeen, T.; Lin, Z.; Gimelshein, N.; Antiga, L. et al. In *Adv. Neural Inf. Process. Syst. 32*; Wallach, H., Larochelle, H., Beygelzimer, A., d'Alché-Buc, F., Fox, E., Garnett, R., Eds.; Curran Associates, Inc., 2019; pp 8024–8035.

(S4) Ball, R. D.; Carrazza, S.; Cruz-Martinez, J.; Del Debbio, L.; Forte, S.; Giani, T.; Iranipour, S.; Kassabov, Z.; Latorre, J. I.; Nocera, E. R. et al. The path to proton structure at one-percent accuracy. *arXiv preprint arXiv:2109.02653* **2021**,

(S5) Egerton, R. F. Limits to the spatial, energy and momentum resolution of electron energy-loss spectroscopy. *Ultramicroscopy* **2007**, *107*, 575–586.

(S6) de la Peña, F.; Prestat, E.; Fauske, V. T.; Burdet, P.; Furnival, T.; Jokubauskas, P.; Nord, M.; Ostasevicius, T.; Lähnemann, J.; MacArthur, K. E. et al. hyperspy/hyperspy: Release v1.6.3. 2021; `https://doi.org/10.5281/zenodo.4923970`.

(S7) Schamm, S.; Zanchi, G. Study of the dielectric properties near the band gap by VEELS:gap measurement in bulk materials. *Ultramicroscopy* **2003**, *96*, 559–564.

(S8) Dimitrievska, M.; Hage, F. S.; Escobar Steinvall, S.; Litvinchuk, A. P.; Stutz, E. Z.; Ramasse, Q. M.; i Morral, A. The Advantage of Nanowire Configuration in Band Structure Determination. *Adv. Funct. Mater.* **2021**, *31*, 2105426.

(S9) Rafferty, B.; Brown, L. M. Direct and indirect transitions in the region of the band gap using electron-energy-loss spectroscopy. *Phys. Rev. B* **1998**, *58*, 10326.

(S10) Rafferty, B.; Pennycook, S. J.; Brown, L. M. Zero loss peak deconvolution for bandgap EEL spectra. *J. Electron Microsc. (Tokyo).* **2000**, *49*, 517–524.

(S11) Gürbulak, B.; cSata, M.; Dogan, S.; Duman, S.; Ashkhasi, A.; Keskenler, E. F. Structural characterizations and optical properties of InSe and InSe:Ag semiconductors grown by Bridgman/Stockbarger technique. *Phys. E Low-dimensional Syst. Nanostructures* **2014**, *64*, 106–111.

(S12) Julien, C. M.; Balkanski, M. Lithium reactivity with III–VI layered compounds. *Mater. Sci. Eng. B* **2003**, *100*, 263–270.

(S13) Rigoult, J.; Rimsky, A.; Kuhn, A. Refinement of the $3R$ γ-indium monoselenide structure type. *Acta Crystallogr. Sect. B* **1980**, *36*, 916–918.

(S14) Lei, S.; Ge, L.; Najmaei, S.; George, A.; Kappera, R.; Lou, J.; Chhowalla, M.; Yamaguchi, H.; Gupta, G.; Vajtai, R. et al. Evolution of the Electronic Band Structure and Efficient Photo-Detection in Atomic Layers of InSe. *ACS Nano* **2014**, *8*, 1263–1272.

(S15) Henck, H.; Pierucci, D.; Zribi, J.; Bisti, F.; Papalazarou, E.; Girard, J.-C.; Chaste, J.; Bertran, F. m. cc.; Le Fèvre, P.; Sirotti, F. et al. Evidence of direct electronic band gap in two-dimensional van der Waals indium selenide crystals. *Phys. Rev. Mater.* **2019**, *3*, 34004.

(S16) Petroni, E.; Lago, E.; Bellani, S.; Boukhvalov, D. W.; Politano, A.; Gürbulak, B.; Duman, S.; Prato, M.; Gentiluomo, S.; Oropesa-Nuñez, R. et al. Liquid-Phase Exfoliated Indium–Selenide Flakes and Their Application in Hydrogen Evolution Reaction. *Small* **2018**, *14*, 1800749.

(S17) van Heijst, S. E.; Mukai, M.; Okunishi, E.; Hashiguchi, H.; Roest, L. I.; Maduro, L.; Rojo, J.; Conesa-Boj, S. Illuminating the Electronic Properties of WS2 Polytypism with Electron Microscopy. *Ann. Phys.* **2021**, *533*, 2000499.

(S18) Maduro, L.; van Heijst, S. E.; Conesa-Boj, S. First-Principles Calculation of Optoelectronic Properties in 2D Materials: The Polytypic WS2 Case. *ACS Phys. Chem. Au* **2022**, in press.
